# Intrinsic endothelial hyperresponsiveness to inflammatory mediators drives acute episodes in models of Clarkson disease

**DOI:** 10.1172/JCI169137

**Published:** 2024-03-19

**Authors:** Ararat J. Ablooglu, Wei-Sheng Chen, Zhihui Xie, Abhishek Desai, Subrata Paul, Justin B. Lack, Linda A. Scott, A. Robin Eisch, Arkadiusz Z. Dudek, Samir M. Parikh, Kirk M. Druey

**Affiliations:** 1 Lung and Vascular Inflammation Section, Laboratory of Allergic Diseases, and; 2 Integrative Data Sciences Section, National Institute of Allergy and Infectious Diseases (NIAID), NIH, Bethesda, Maryland, USA.; 3 Division of Medical Oncology, Mayo Clinic, Rochester, Minnesota, USA.; 4 Division of Nephrology, Departments of Internal Medicine and Pharmacology, University of Texas Southwestern Medical Center, Dallas, Texas, USA.

**Keywords:** Vascular biology, Endothelial cells

## Abstract

Clarkson disease, or monoclonal gammopathy–associated idiopathic systemic capillary leak syndrome (ISCLS), is a rare, relapsing-remitting disorder featuring the abrupt extravasation of fluids and proteins into peripheral tissues, which in turn leads to hypotensive shock, severe hemoconcentration, and hypoalbuminemia. The specific leakage factor(s) and pathways in ISCLS are unknown, and there is no effective treatment for acute flares. Here, we characterize an autonomous vascular endothelial defect in ISCLS that was recapitulated in patient-derived endothelial cells (ECs) in culture and in a mouse model of disease. ISCLS-derived ECs were functionally hyperresponsive to permeability-inducing factors like VEGF and histamine, in part due to increased endothelial nitric oxide synthase (eNOS) activity. eNOS blockade by administration of *N*(γ)-nitro-l-arginine methyl ester (l-NAME) ameliorated vascular leakage in an SJL/J mouse model of ISCLS induced by histamine or VEGF challenge. eNOS mislocalization and decreased protein phosphatase 2A (PP2A) expression may contribute to eNOS hyperactivation in ISCLS-derived ECs. Our findings provide mechanistic insights into microvascular barrier dysfunction in ISCLS and highlight a potential therapeutic approach.

## Abstract 2

An autonomous endothelial defect characterized by cytokine hypersensitivity underlies vascular leakage in Clarkson disease.

## Introduction

The initial presentation of Clarkson disease, or monoclonal gammopathy–associated idiopathic systemic capillary leak syndrome (ISCLS), is frequently complicated by multiple organ dysfunction syndrome (MODS), rhabdomyolysis, and intravascular thrombosis (1, 2). Compartment syndrome may occur in the extremities due to excessive administration of i.v. fluids, frequently necessitating fasciotomies and/or limb amputation. Vascular leakage ultimately recedes spontaneously, typically after several days, which is followed by the mobilization of extravasated fluids into the circulation in a “post-leak” phase. During this period, patients are at high risk for flash pulmonary edema due to cardiac dysfunction. Between episodes, patients show no overt pathological phenotype. Mortality during ISCLS flares approaches 30%, in part because no acute intervention has been proven to shorten the duration of episodes or prevent complications (1). By contrast, monthly prophylaxis with high-dose intravenous immunoglobulins (IVIGs) substantially reduces the frequency and severity of ISCLS flares and increases survival (3–5).

The mechanism by which IVIG prevents ISCLS relapse is unknown. Most ISCLS flares are triggered by antecedent infections such as viral upper respiratory infections (including COVID-19), suggesting a role for inflammation in the induction of vascular leakage (1, 6). However, extensive proteomics profiling of acute ISCLS sera has not yet uncovered unique humoral factor(s) that may trigger attacks (7, 8). More than 90% of patients with ISCLS have a monoclonal gammopathy of unknown significance (MGUS, typically IgG κ), but its role in disease pathogenesis is unknown (5).

Although fewer than 500 cases of ISCLS have been described in the medical literature (9), we have assembled the world’s largest ISCLS registry (>80 patients with a confirmed diagnosis), and our previous studies point to microvascular dysregulation in this disease. Patients challenged intradermally with permeability provocateurs (morphine or histamine) have increased vascular leakage compared with healthy controls, as evidenced by the increased size of skin wheals due to localized edema (10). Blood-outgrowth endothelial cells (BOECs) expanded from patients with asymptomatic ISCLS have gene expression patterns that differ significantly from those in cells from healthy controls (8, 11). In a mouse model of ISCLS induced by systemic histamine challenge, SJL/J mice are uniquely susceptible to vascular leakage and mortality compared with most other inbred strains (10, 12). This autosomal recessive trait, termed *Histh*, maps to a quantitative trait locus on Chr6 that is syntenic to the locus most closely aligned with ISCLS in a human genomic association study (Chr3.25p) (13).

On the basis of these findings suggestive of vascular hypersensitivity, we hypothesized that acute ISCLS flares are initiated by an autonomous endothelial defect characterized by exaggerated barrier dysfunction in response to proinflammatory mediators. To test this, we examined the morphology and functional behavior of the microvasculature of patients in situ and in BOECs ex vivo. Patient-derived ECs were hyperresponsive to several mediators of permeability in an eNOS-dependent fashion. Inhibition of eNOS ameliorated vascular leakage in the SJL/J mouse model of ISCLS, suggesting a therapeutic approach to acute disease flares.

## Results

### The endothelial response to histamine is exaggerated in patients with ISCLS.

**Figure 1:**
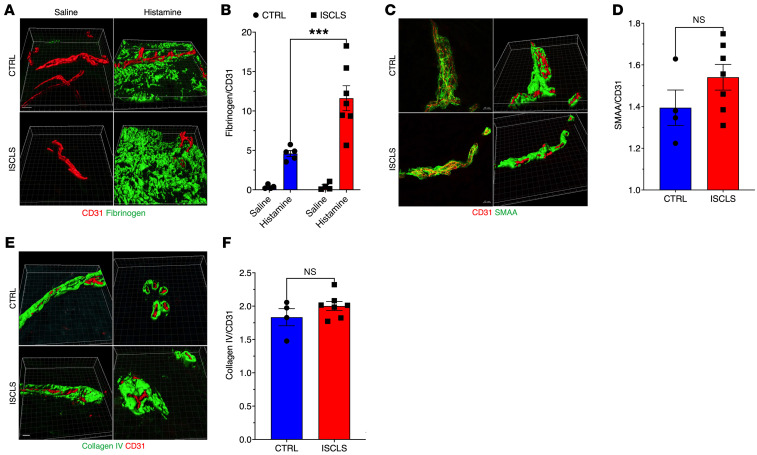
Increased histamine-induced vascular leakage in ISCLS skin. (**A**) 3D rendering of fibrinogen (green) and CD31 (red) immunostaining in representative skin biopsies obtained after intradermal challenge with saline or histamine. Scale bar: 10 μm. Original magnification, ×63. (**B**) Ratio of fibrinogen/CD31 area (μm^2^). Data indicate the mean ± SEM. *n* = 7 ISCLS; *n* = 5 controls (CTRL). ***P = 0.0006, by 2-way ANOVA with Šidák’s multiple comparisons. (**C** and **D**) α-Smooth muscle actin (SMAA, green) or CD31 (red) immunostaining (left panels) and 3D rendering (right panels) in representative skin biopsies (**C**) and quantification of the SMAA/CD31 ratio (**D**). Data indicate the mean ± SEM. *n* = 4 ISCLS; *n* = 7 controls. NS, by unpaired, 2-tailed Student’s *t* test. Scale bar: 10 μm. Original magnification, ×63. (**E** and **F**) Collagen type IV (green) or CD31 (red) immunostaining in representative skin biopsies (**E**) and quantification of the collagen IV/CD31 ratio (**F**). Data indicate the mean ± SEM. *n* = 4 ISCLS; *n* = 7 controls. NS, by unpaired, 2-tailed Student’s *t* test. Scale bar: 10 μm. Original magnification, ×63.

Intradermal challenge with unrelated leak-inducing agents (histamine and morphine) elicits significantly more focal skin edema in patients with ISCLS than in controls (10). To determine the cellular and molecular mechanisms underlying this observation, we used immunofluorescence to quantify extravasation of the serum protein fibrinogen in fixed skin biopsies from challenged patients (14). Demographic information about the patients and healthy controls is shown in Supplemental Table 1; supplemental material available online with this article; https://doi.org/10.1172/JCI169137DS1 The ISCLS group was significantly older (albeit with a similar range) and had more male participants and more White participants than did the control group. A solid majority of the patients with ISCLS had MGUS, whereas none of the controls did. The endothelial marker CD31 was used to quantify the vascular area of the skin. As expected, little to no fibrinogen immunostaining was observed in saline-challenged skin, whereas we detected abundant extravascular fibrinogen at the histamine-challenged sites (Figure 1A). The extravascular fibrinogen^+^ area was significantly greater in histamine-challenged skin of patients with ISCLS compared with that of healthy controls (Figure 1, A and B). Baseline serum fibrinogen levels did not differ between the groups (Supplemental Figure 1). Since histamine acts directly on H1 receptors expressed on ECs (15), these results suggested an increased functional response to histamine in the ISCLS endothelium.

Neither vasculitis nor aberrant angiogenesis has been observed consistently in biopsies of skin or skeletal muscle taken from patients during ISCLS flares (9, 16). We did not find evidence of cellular inflammation or other histological abnormalities in the skin of asymptomatic patients by light microscopy (Supplemental Figure 2). Dropout of pericytes, smooth muscle–like cells that line microvessels, has been associated with endothelial hyperpermeability (17, 18). However, we detected equivalent pericyte coverage (immunoreactive smooth muscle α actin) of microvessels in ISCLS and control biopsies (Figure 1, C and D). Likewise, aberrant expression or interaction of extracellular matrix components such as collagen IV with integrins expressed on ECs regulates vascular barrier function (19, 20). However, we found that collagen IV immunostaining around vessels in ISCLS and healthy control skin did not differ from one another (Figure 1, E and F). These findings further support the hypothesis that an exaggerated response to proinflammatory mediators, rather than underlying structural defects in the microvasculature, probably underlies vascular leakage during acute ISCLS flares.

### Disruption of adherens junctions in ISCLS dermal microvascular endothelium.

**Figure 2:**
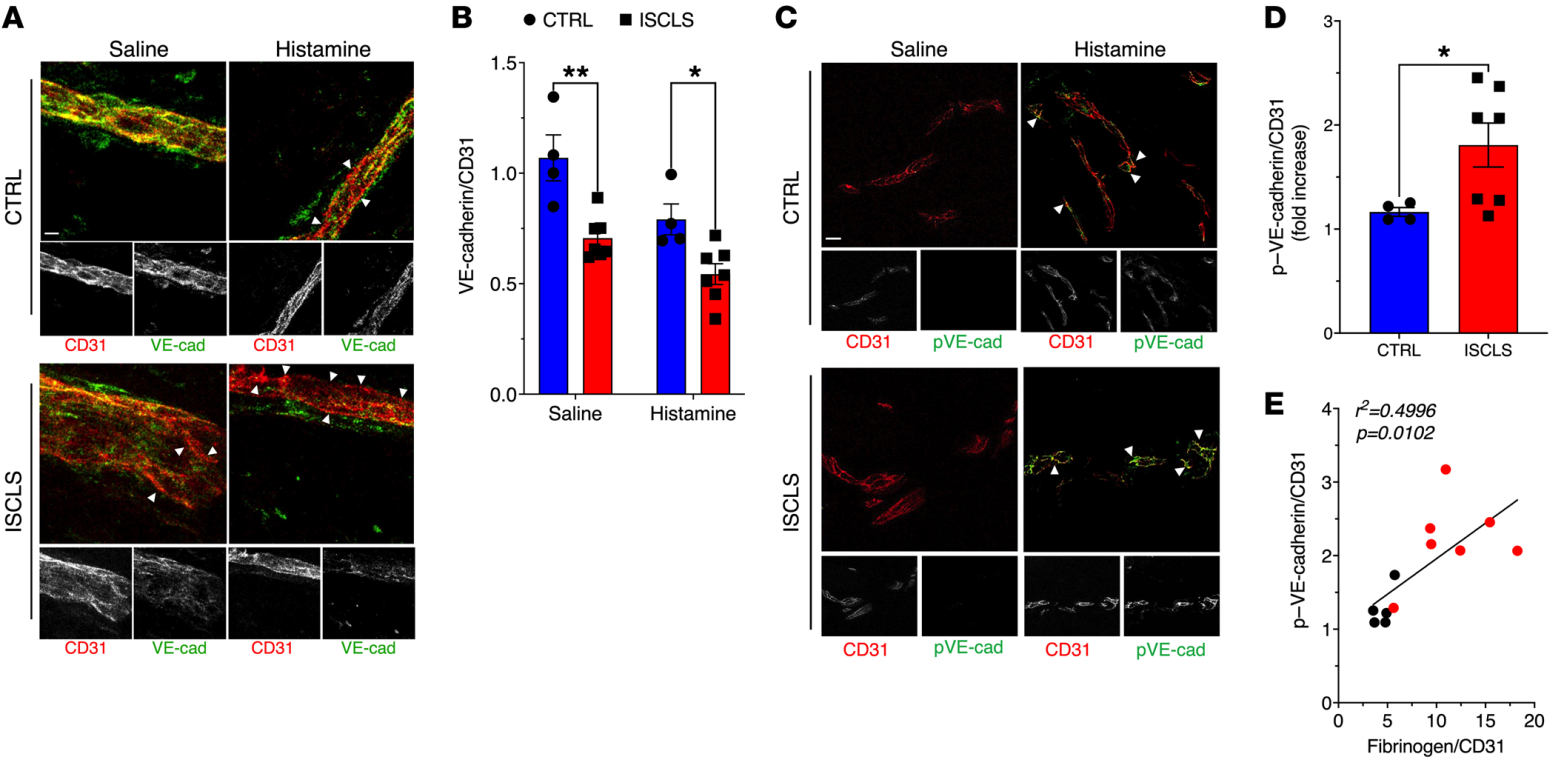
Adherens junction protein expression in dermal microvasculature. (**A**) VE-cadherin (VE-cad; green) or CD31 (red) immunostaining in representative skin biopsies; arrowheads indicate areas of decreased VE-cadherin expression (red), which is reflected in the black/white panels of individual immunostains (lower panels). (**B**) Ratio of VE-cadherin/CD31 immunostaining. Data indicate the mean **±** SEM. *n* = 7 ISCLS; *n* = 4 controls. *P = 0.01 and **P = 0.001, by 2-way ANOVA with Šidák’s multiple comparisons. (**C**) p–VE-cadherin^Tyr685^ (green, arrowheads) and CD31 (red) immunostaining in representative skin biopsies; black/white panels show the corresponding individual stains. (**D**) p–VE-cadherin/CD31 immunostaining (μm^2^) in histamine-challenged skin biopsies. Data indicate the mean ± SEM. *n* = 7 ISCLS; *n* = 4 controls. *P = 0.02, by Mann-Whitney *U* test. (**E**) Pearson’s correlation of p–VE-cadherin immunostaining with fibrinogen extravasation. Scale bars: 10 μm. Original magnification, ×63.

Application of acute, but not convalescent, ISCLS sera to normal dermal microvascular ECs induces transient barrier disruption — but not apoptosis, injury, or activation — through mechanisms that include disruption of adhesion junctions and cytoskeletal rearrangements that promote EC contraction (21, 22). To determine the molecular mechanisms involved in histamine-evoked vascular leakage in ISCLS skin, we quantified the expression of VE-cadherin, an essential mediator of endothelial intercellular adhesion, in skin biopsies. As expected, histamine challenge reduced VE-cadherin expression in skin of both groups compared with skin injected with saline alone (Figure 2A). However, the VE-cadherin^+^ area in skin microvasculature was already lower at baseline in patients with ISCLS than in controls and decreased further following histamine challenge (Figure 2B).

Proinflammatory mediators including histamine, bradykinin, and VEGF induce Src-mediated tyrosine phosphorylation of VE-cadherin on Tyr^685^, which is required for the internalization and dissolution of adherens junctions (23). To assess the extent of VE-cadherin phosphorylation in skin biopsies, we assessed phosphorylated VE-cadherin (p-Tyr^685^) in skin biopsies by immunofluorescence. We first evaluated the specificity of a VE-cadherin (p-Tyr^685^) antibody used previously for immunostaining (24). We detected substantially reduced amounts of phosphorylated and total VE-cadherin in lysates from BOECs transfected with a VE-cadherin–targeted siRNA immunoprecipitated with VE-cadherin antibody compared with controls, confirming its specificity (Supplemental Figure 3). Using this antibody, we detected a significant increase in VE-cadherin (p-Tyr^685^) immunostaining in histamine-challenged dermal blood vessels compared with those treated with saline, and the increase in ISCLS skin was nearly double that seen in healthy controls (Figure 2, C and D). Moreover, there was a significant correlation between VE-cadherin (p-Tyr^685^) and fibrinogen extravasation (Figure 2E). Our findings thus far suggested that the dermal microvasculature in ISCLS had impaired barrier function at homeostasis and after histamine challenge due to reduced VE-cadherin expression.

### ISCLS-derived ECs exhibit durable hyperresponsiveness in vitro.

To determine whether the increased permeability of ISCLS dermal microvasculature observed in situ was due to an autonomous endothelial defect, we systematically characterized the functional responses of patient-derived ECs to various mediators ex vivo. For these studies, we used BOECs expanded from the blood of patients and healthy controls over multiple passages. BOECs had an endothelial morphology when visualized by light microscopy, formed confluent monolayers (11) (Supplemental Figure 4A), and were uniformly CD31^+^CD45^–^, as expected (25). CD31 expression was comparable in BOECs from patients with ISCLS and those from healthy controls (Supplemental Figure 4, B–D).

**Figure 3:**
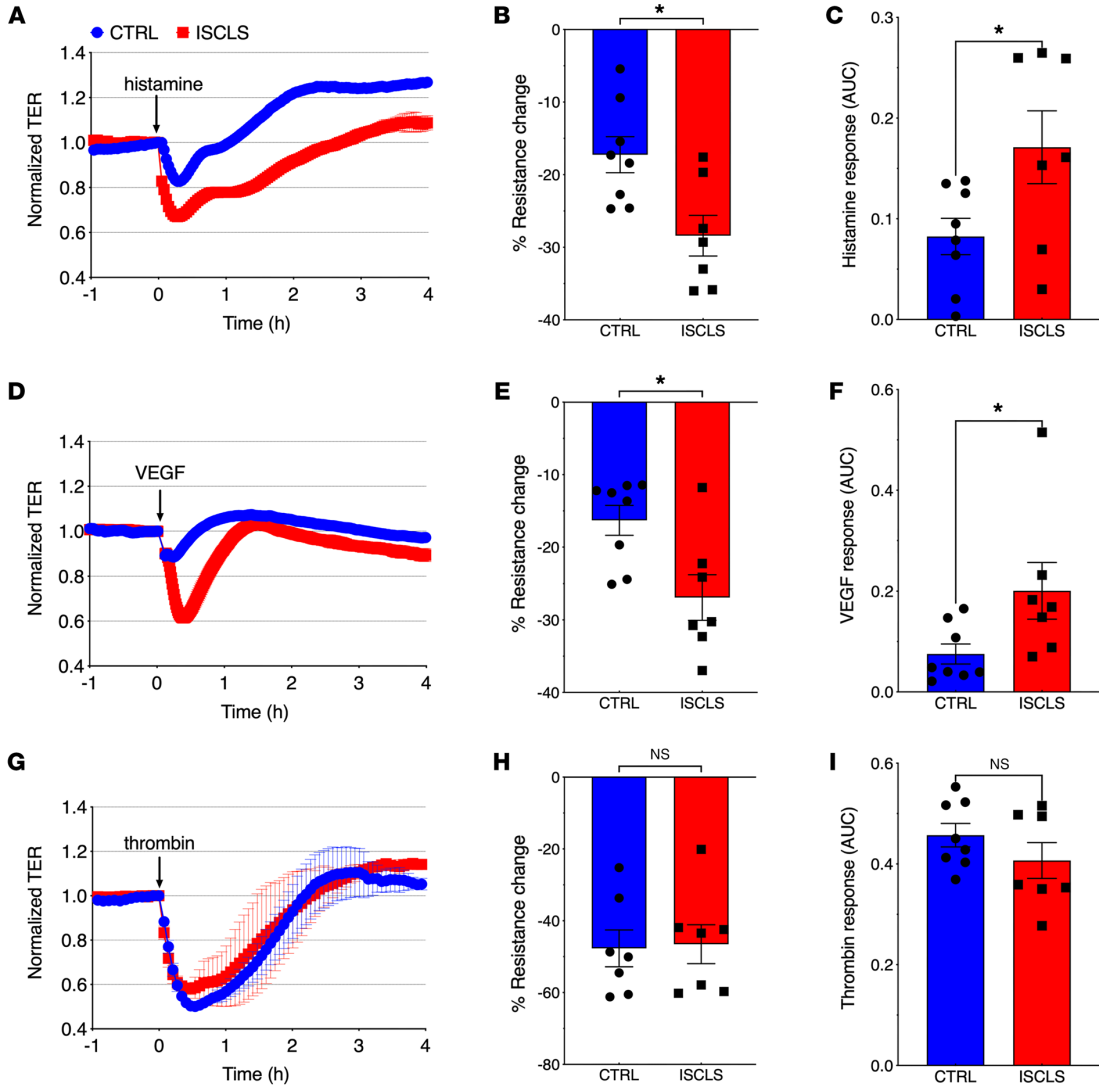
Hyperresponsiveness of ISCLS-derived ECs in vitro. Representative TER over time in BOEC monolayers stimulated with histamine (20 μM) (**A**), VEGF (100 ng/mL) (**D**), or thrombin (0.05 U/mL) (**G**). Data indicate the mean ± SD. Arrows indicated the time of agonist addition. (**B**, **E**, and **H**) Maximum decrease in TER elicited by the respective agonists. Data indicate the mean ± SEM. *n* = 7 ISCLS; *n* = 8 controls. *P = 0.01, by unpaired, 2-tailed Student’s *t* test. (**C**, **F**, and **I**) The AUC is shown for each agonist. Data indicate the mean ± SEM. *n* = 7 ISCLS; *n* = 8 controls. *P = 0.04, by unpaired, 2-tailed Student’s *t* test (**C**) and ^#^*P* = 0.01, by Mann Whitney *U* test (**F**).

We used electric cell impedance sensing (ECIS) to assess transendothelial resistance (TER) in real time through measurements of paracellular passage of low-frequency (4 kHz) current. Whereas baseline resistance was comparable in ISCLS-derived or control BOECs, TER decreased significantly more and recovered more slowly in ISCLS BOECs stimulated with histamine (Figure 3, A–C) or VEGF (Figure 3, D–F). Thrombin induced a comparable drop in resistance and recovery in ISCLS and control BOECs, even at submaximal concentrations (Figure 3, G–I).

**Figure 4:**
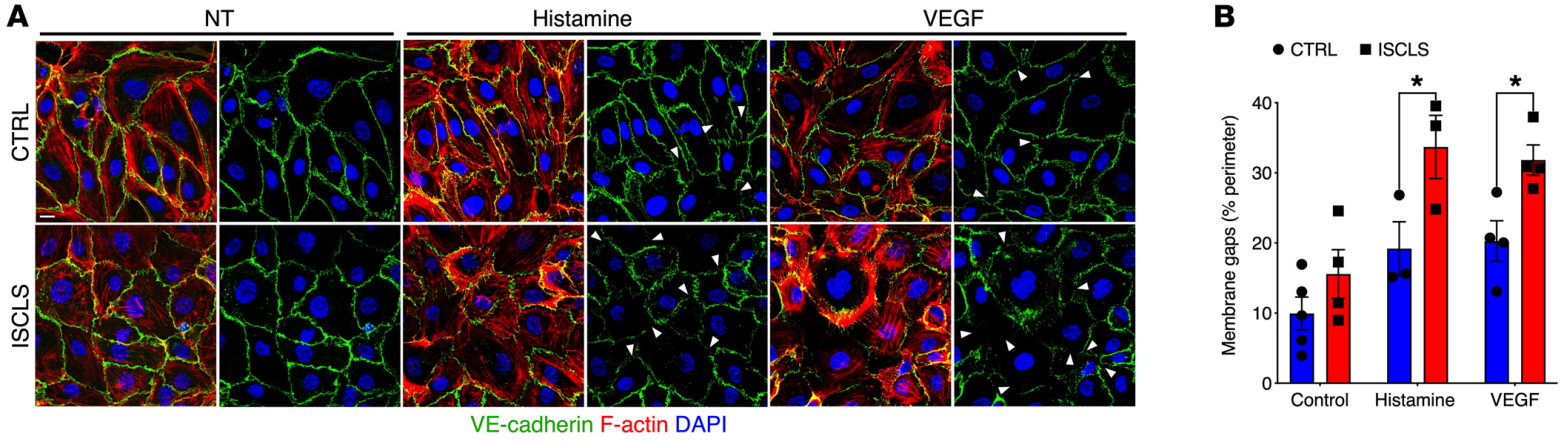
Structural correlates of impaired barrier function in BOECs. (**A**) Representative images of BOECs at homeostasis (nontreated [NT]) or stimulated with histamine (20 μM) or VEGF (100 ng/mL) for 15 minutes and then stained with VE-cadherin antibody (green), phalloidin (red), or DAPI (blue). Arrowheads indicate areas of membrane disruption. Scale bar: 15 μm. Original magnification, ×63. (**B**) Quantification of membrane disruption as a percentage of the cell perimeter. Data indicate the mean ± SEM. *n* = 2–3 donors/group analyzed in 3–5 independent experiments. *P < 0.04, by2-way ANOVA with Šidák’s multiple comparisons.

Next, we examined dynamic changes in endothelial morphology that might account for the functional hyperresponsiveness. Untreated control and ISCLS BOECs had comparable membrane-associated VE-cadherin expression and cortical actin (Figure 4A). Histamine or VEGF elicited paracellular gap formation, decreased VE-cadherin localization at intercellular junctions, and F-actin rearrangements (reduced cortical actin and increased planar actin stress fibers) compared with untreated cells, as expected. In line with the responses of ISCLS skin microvasculature, the loss of membrane-associated VE-cadherin expression was significantly more prominent in histamine- or VEGF-treated ISCLS BOECs than in control cells (Figure 4B). These findings support the hypothesis that functional defects in the ISCLS endothelium in response to permeability-inducing factors may be sufficient to account for the vascular hyperresponsiveness observed in situ.

### Increased eNOS phosphorylation in ISCLS-derived BOECs.

**Figure 5:**
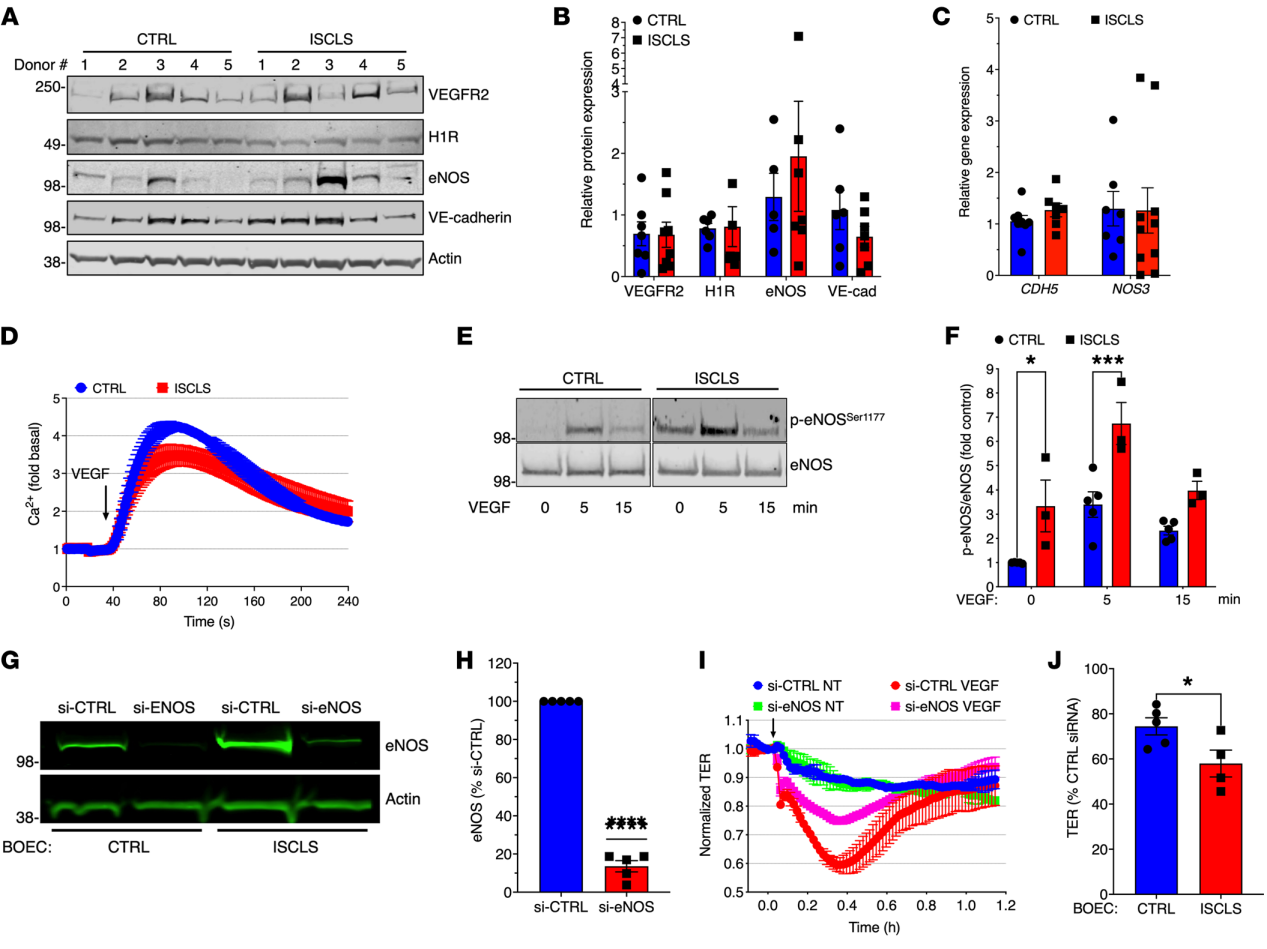
Hyperresponsiveness of ISCLS BOECs is eNOS dependent. (**A**) Representative immunoblot of relevant receptors or signaling proteins in BOEC cell lysates (*n* **=** 5 donors/group). (**B**) Quantification of relative protein expression. Data indicate the mean ± SEM. *n* = 6–9 donors/group. NS, by 2-way ANOVA with Šidák’s multiple comparisons. (**C**) Relative *CDH5* or *NOS3* expression in BOECs evaluated by quantitative PCR (qPCR) (normalized to *Actb* and/or *GAPDH*). Data indicate the mean ± SEM. *n* = 6–10 donors/group. NS, by Mann-Whitney *U* test. (**D**) Relative intracellular Ca^2+^ concentrations in control (blue) or ISCLS-derived (red) BOECs stimulated with VEGF (100 ng/mL). Data indicate the mean ± SEM. *n* = 2–3 donors/group analyzed in 4–5 independent experiments. (**E**) Representative immunoblot showing p-eNOS^Ser1177^ and total eNOS levels in lysates from BOECs stimulated with VEGF and immunoprecipitated with anti-eNOS antibody. (**F**) Quantification of the p-eNOS/eNOS ratio. Data indicate the mean ± SEM of 3–5 donors/group analyzed in 5 independent experiments. *P = 0.01 and ***P = 0.0008, by 2-way ANOVA with Šidák’s multiple comparisons. (**G** and **H**) Representative immunoblot (**G**) and quantification (**H**) of eNOS/actin in eNOS siRNA–transfected BOECs. Data indicate the mean ± SEM of 5 independent experiments. ****P < 0.0001, by 1-sample Student’s *t* test. (**I**) Representative TER in control or eNOS siRNA–transfected BOECs left untreated (blue and green) or stimulated with VEGF (red and magenta). (**J**) Maximum decrease in VEGF-induced TER from *t* = 0 as a percentage of the control siRNA response. Data indicate the mean ± SEM. *n* = 4–5 donors/group analyzed in 3–5 independent experiments. *P = 0.04, by unpaired, 2-tailed Student’s *t* test.

We next investigated the mechanisms contributing to increased permeability of the ISCLS endothelium. Expression of VEGFA (VEGFR2) and histamine (H1) receptors, key signaling proteins (eNOS, VE-cadherin), or transcripts for *CDH5* (encoding VE-cadherin) or *NOS3* (encoding eNOS) was comparable in control and ISCLS BOECs (Figure 5, A–C). These results suggested that dysregulation of downstream intracellular signaling pathways might underlie the amplified functional responses of ISCLS-derived BOECs. Because we have previously detected elevated VEGF levels in acute ISCLS sera (21, 26), we focused specifically on the mechanisms underlying VEGF-induced hyperpermeability. VEGFR2-mediated signaling in several types of ECs hinges on activation of multiple effectors including Src, phosphatidylinositol 3-kinase (PI3K), and phospholipase Cγ1 (PLCγ1) (27). VEGF-induced increases in cytosolic Ca^2+^ (due to PLCγ1 activation) were similar in control and ISCLS BOECs (Figure 5D). Ca^2+^ flux elicited by histamine or ionomycin was also equivalent in ISCLS and control BOECs (Supplemental Figure 5, A and B). Unexpectedly, basal Akt phosphorylation (indicative of PI3K activation) was prominent in both control and ISCLS-derived BOECs to a comparable extent, and VEGF treatment did not elicit a significant increase in p-Akt in either cell type (Supplemental Figure 6, A and B). Although the mechanisms underlying these results require further study, they nonetheless suggested that increased PI3K activation did not contribute to the hyperresponsiveness of ISCLS-derived BOECs to VEGF.

To identify other perturbations in ISCLS cells, we conducted a phosphoproteomics screen. Among the most differentially phosphorylated proteins in VEGF-stimulated, ISCLS-derived BOECs compared with controls were eNOS (p-Ser^1177^, 2.3-fold higher), AMPK (p-Thr^172^), and β-catenin (Supplemental Figure 7). In immunoblots of BOEC lysates from individual subjects, baseline and VEGF-stimulated eNOS phosphorylation levels were significantly increased in ISCLS BOECs compared with controls (Figure 5, E and F).

Previous studies have demonstrated that eNOS activation is critical for histamine and VEGF-induced vascular leakage in ECs in vitro and in mice (28). To determine the role of increased eNOS phosphorylation in the impaired barrier function of ISCLS-derived BOECs, we transfected cells with an eNOS-specific siRNA, which reduced eNOS protein levels by more than 80% compared with cells transfected with a control siRNA (Figure 5, G and H). Although knockdown of eNOS attenuated the VEGF-evoked decrease in TER in both control and ISCLS-derived BOECs (Figure 5I), the responses of ISCLS-derived cells were inhibited to a significantly greater extent (Figure 5J). These findings suggest that the hyperresponsiveness of ISCLS ECs to VEGF was uniquely eNOS dependent.

### eNOS blockade mitigates vascular leakage in a mouse model of ISCLS.

Like adult patients with ISCLS, aged SJL/J mice (>6 months of age) have no overt baseline vascular phenotype but are unusually susceptible to histamine challenge. Low doses of histamine (2.5 mg/kg) elicit vascular leakage in SJL/J mice, most prominently in peripheral tissues like skin and skeletal muscle (10). In contrast, much higher doses of histamine (1–2.5 log-fold) are typically required to induce vascular leakage in most inbred mouse strains including C57BL/6 (29–32). In our previous study, we observed that a high proportion of SJL/J mice die within 30 minutes of systemic challenge with histamine at doses as low as 10 mg/kg, unlike what was observed in more than 20 of the other strains tested (10, 12). The genetic and phenotypic similarity of the Histh trait to the vascular hypersensitivity observed in patients with ISCLS suggests shared pathophysiological mechanisms.

**Figure 6:**
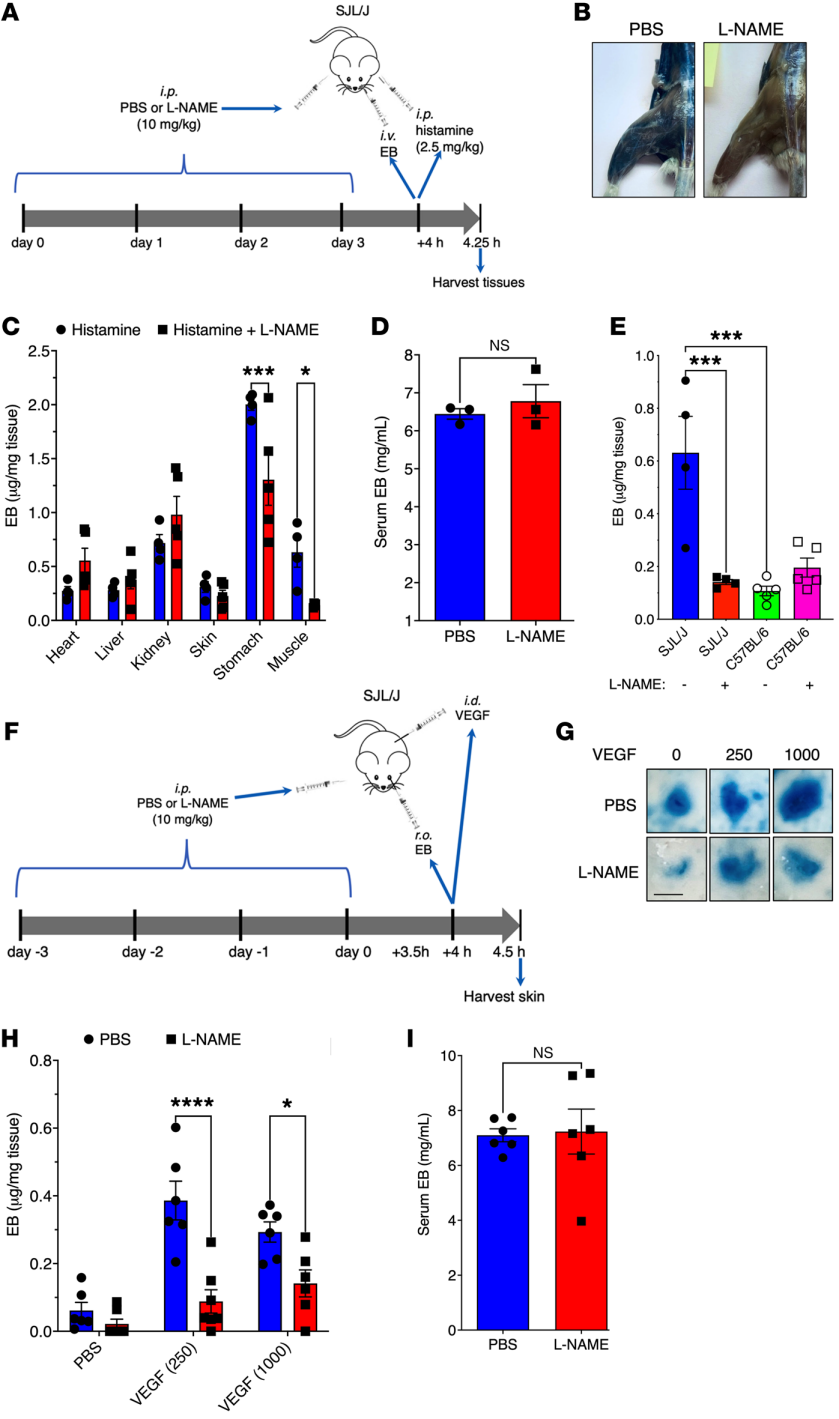
Effect of eNOS blockade in a mouse model of ISCLS. (**A**) Analysis of histamine-induced systemic vascular leakage in SJL/J mice. (**B**) Representative images of EB extravasation in the legs of mice pretreated with l-NAME (10 mg/kg) or PBS and challenged with histamine (2.5 mg/kg i.p.). (**C** and **D**) Relative EB quantities in organs (**C**) or serum (**D**) from SJL/J mice challenged with histamine systemically. Data indicate the mean ± SEM. *n* = 3–5 mice/group. *P = 0.04 and ***P = 0.001, by 2-way ANOVA with Šidák’s multiple comparisons (**C**); NS, by Mann-Whitney *U* test (**D**). (**E**) EB content in muscle from the respective mice pretreated or not with l-NAME and challenged with histamine systemically. Data indicate the mean ± SEM. *n* = 4–5 mice/group. ***P < 0.0009, by 1-way ANOVA, Tukey’s multiple comparisons. (**F**) Analysis of vascular leakage in skin following intradermal challenge with VEGF. (**G**) Representative images of EB in skin challenged as indicated. Scale bar: 5 μm. (**H** and **I**) Relative EB quantities in skin (**H**) and serum (**I**) of mice challenged with VEGF intradermally. Data indicate the mean ± SEM. *n* = 6–7 mice/group. *P = 0.01 and ****P < 0.0001, by 2-way ANOVA with Šidák’s multiple comparisons (**H**); NS, by Mann-Whitney *U* test (**I**).

To characterize the contribution of eNOS to vascular leakage in this ISCLS model, we treated aged SJL/J mice with the competitive eNOS inhibitor l-NAME prior to systemic challenge with low doses of histamine and measured Evans blue (EB) extravasation in peripheral tissues with a specific focus on skeletal muscle, which is the predominant site of vascular leakage in patients (Figure 6A). l-NAME prophylaxis significantly attenuated EB extravasation in muscle and stomach of histamine-challenged mice compared with vehicle alone (Figure 6, B and C). Serum EB levels were comparable in PBS- or l-NAME–pretreated mice (Figure 6D). By contrast, this low dose of histamine elicited significantly less EB extravasation in muscle from histamine-resistant mice (aged-matched C57BL/6J) (12), and l-NAME pretreatment had no effect on the response (Figure 6E and Supplemental Figure 8). To evaluate the effect of l-NAME on the in vivo responses to an ISCLS-related cytokine, we measured EB extravasation in the skin of SJL/J mice after intradermal injection with VEGF (Figure 6F). l-NAME pretreatment significantly reduced EB content in VEGF-challenged skin compared with controls (Figure 6, G and H) but not in serum (Figure 6I). These findings suggest a unique and important role of eNOS in the pathophysiology of vascular leakage in the Histh model of ISCLS.

### Mechanisms underlying increased eNOS activity in ISCLS ECs.

**Figure 7:**
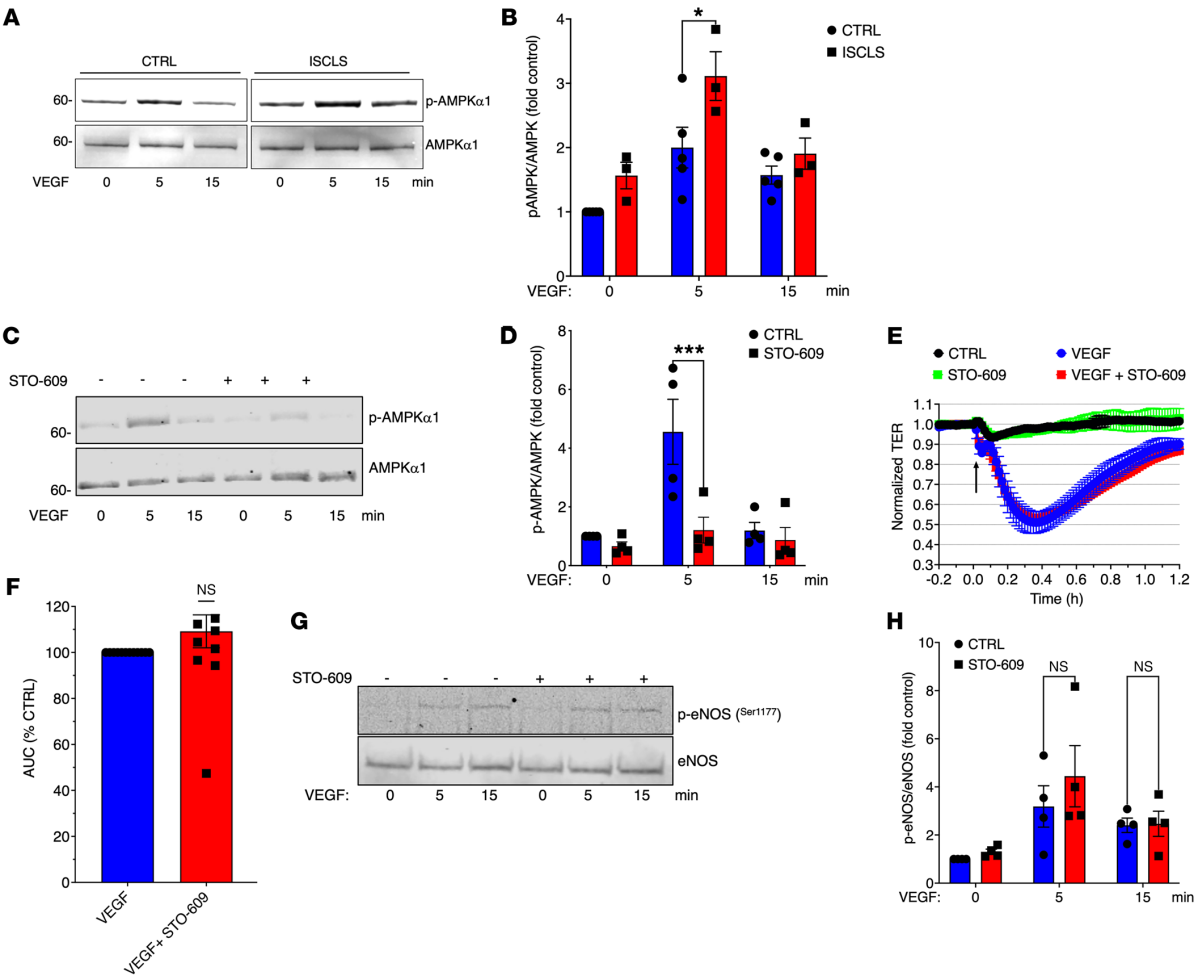
Role of AMPK in eNOS hyperphosphorylation in ISCLS-derived BOECs. (**A** and **B**) Representative blot (**A**) and quantification of p-AMPK/AMPK in BOECs left untreated or stimulated with VEGF (100 ng/mL) (**B**). Data indicate the mean ± SEM. *n* = 3–4 donors/group. *P = 0.01, by 2-way ANOVA with Šidák’s multiple comparisons. (**C** and **D**) Representative blot (**C**) and quantification (**D**) of p-AMPK/AMPK in BOECs pretreated with STO-609 (12 μM for 6 hours) or vehicle and stimulated with VEGF (100 ng/mL) for the indicated durations. Data indicate the mean ± SEM. *n* = 2 donors/group analyzed in 4 independent experiments. ***P = 0.0009, by 2-way ANOVA with Šidák’s multiple comparisons. (**E** and **F**) TER (**E**) and AUC (**F**) in BOECs pretreated with vehicle or STO-609. *n* = 2 donors/group analyzed in 4 independent experiments. NS, by 1-sample Student’s *t* test. (**G**) Representative blot (**G**) and quantification (**H**) of p-eNOS/eNOS in BOECs left untreated or pretreated with STO-609 and stimulated with VEGF (100 ng/mL). Data indicate the mean ± SEM. *n* = 3–4 donors/group. NS, by 2-way ANOVA with Tukey’s multiple comparisons.

We explored the potential causes of increased VEGF-induced eNOS phosphorylation in ISCLS-derived BOECs. VEGF activates several kinases that have the capacity to phosphorylate eNOS, including Akt and AGC protein kinases (e.g., AMPK) (28). Although the contribution of Akt to the hyperreactivity of ISCLS-derived BOECs is unclear, as noted above, AMPK phosphorylation was significantly increased in VEGF-treated ISCLS BOECs compared with control cells (Figure 7, A and B).

Although AMPK has a well-established function as a sensor of increased intracellular AMP levels, previous studies of its role in endothelial permeability have yielded conflicting results (33, 34). Currently available chemical inhibitors of AMPK (e.g., compound C) display considerable nonspecificity in cells (35, 36), and we could not achieve robust knockdown of AMPK protein expression in BOECs by RNAi. AMPK is activated by several upstream kinases including Ca^2+^-calmodulin–dependent kinase kinase β (CAMKKβ). To clarify the effect of AMPK on hyperresponsiveness due to eNOS activation in BOECs, we exposed cells to STO-609, a specific inhibitor of CAMKKβ. Although pretreatment with STO-609 completely blocked VEGF-induced AMPK phosphorylation (Figure 7, C and D), it had no effect on VEGF-induced barrier dysfunction (Figure 7, E and F) or eNOS phosphorylation (Figure 7, G and H) in either ISCLS-derived or control BOECs. These findings suggest that AMPK did not contribute to eNOS hyperactivation in ISCLS-derived BOECs.

**Figure 8:**
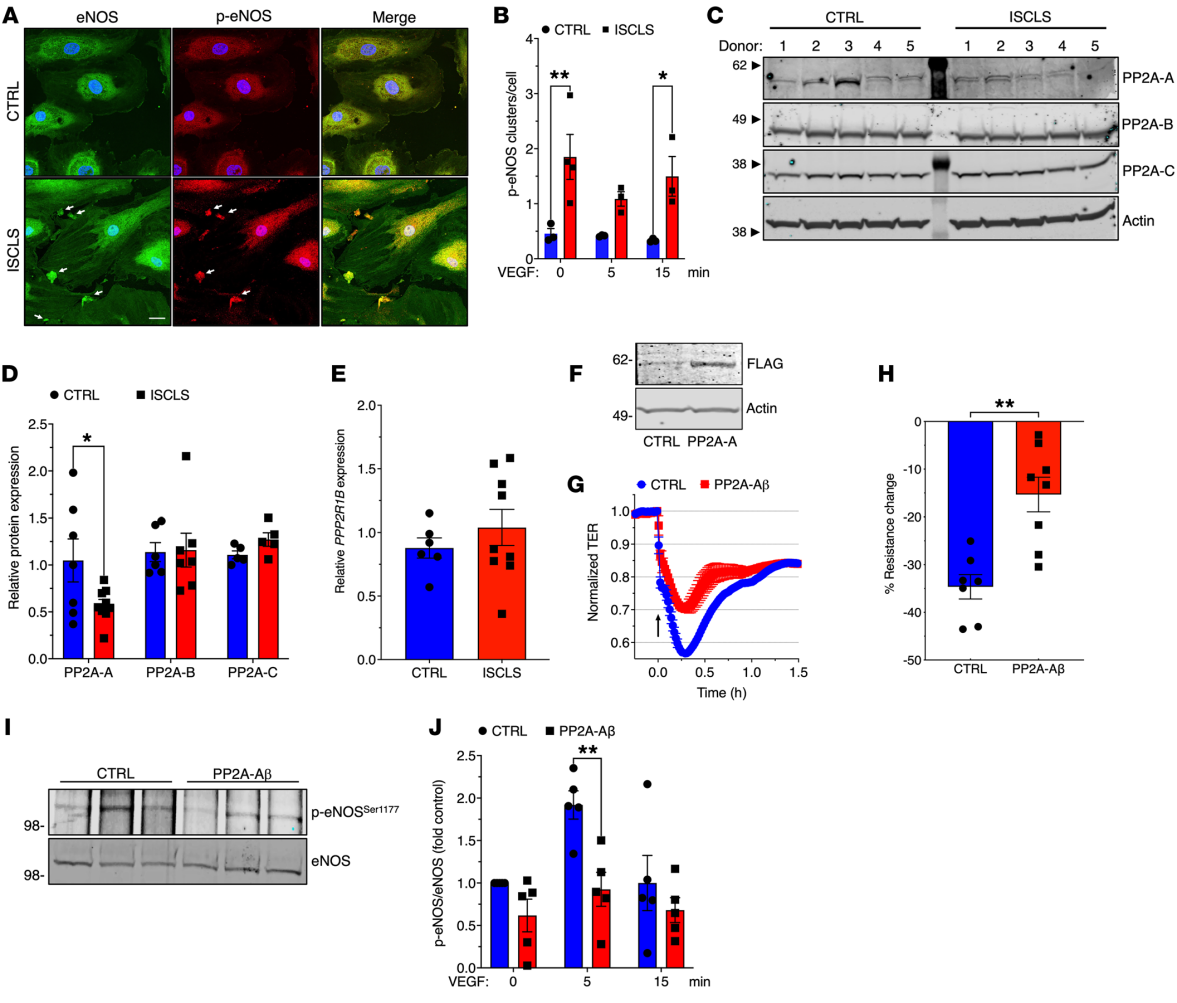
Aberrant eNOS localization and PP2A expression in ISCLS-derived BOECs. (**A**) BOECs stained with anti–p-eNOS (left, green) or anti-eNOS (middle, red) antibodies and DAPI. Arrows denote eNOS clusters. Scale bar: 20 μm. (**B**) Numbers of eNOS clusters/cell. Data indicate the mean ± SEM. *n* = 3 cell lines/group. *P = 0.03 and **P = 0.006, by 2-way ANOVA with Šidák’s multiple comparisons. (**C**) Representative immunoblot of PP2A subunits in BOEC lysates (*n* = 5 donors/group). (**D**) Quantification of relative protein expression. Data indicate the mean ± SEM. *n* = 7–9 donors/group. *P = 0.03, by 2-way ANOVA with Šidák’s multiple comparisons. (**E**) Relative *PPP2R1B* expression in BOECs evaluated by qPCR (normalized to *GAPDH*). Data indicate the mean ± SEM. *n* = 6–9 donors/group. NS, by unpaired, 2-tailed Student’s *t* test. (**F**) Representative immunoblot of FLAG-PP2A-Aβ in BOECs transfected with the respective lentiviruses. (**G** and **H**) Representative TER (**G**) and maximum decrease in VEGF-induced TER (**H**) from *t* = 0 (arrow) in BOECs infected with control or FLAG–PP2A-β–encoding lentivirus. Data indicate the mean ± SEM. *n* = 2 donors/group analyzed in 4 independent experiments. **P = 0.001, by unpaired, 2-tailed Student’s *t* test. (**I** and **J**). Representative blot (**I**) and quantification (**J**) of p-eNOS/eNOS in control versus FLAG–PP2A-β–overexpressing BOECs stimulated with VEGF (100 ng/mL). Data indicate the mean ± SEM. *n* = 5 independent experiments. **P = 0.004, by 2-way ANOVA with Šidák’s multiple comparisons.

Because we were unable to clearly identify causative perturbations in the most well-characterized VEGF-stimulated signaling pathways upstream of eNOS in ISCLS-derived BOECs, we considered the possibility of aberrant subcellular eNOS localization. Published studies have demonstrated that eNOS/p-eNOS localize predominantly at the cytoplasmic face of the Golgi in ECs, with a smaller fraction at the plasma membrane (PM) (37, 38). In both ISCLS-derived and control BOECs, we observed p-eNOS/eNOS immunostaining primarily in the perinuclear region, consistent with Golgi localization, with a smaller fraction at the PM (Figure 8, A and B). However, there were large clusters of PM-associated p-eNOS/eNOS in ISCLS-derived BOECs in the presence or absence of VEGF stimulation that were absent in control cells (Figure 8, A and B, and Supplemental Videos 1 and 2).

Beyond a potential role of eNOS mislocalization, we hypothesized that aberrant expression and/or function of intracellular regulators of eNOS activity might also contribute to the observed functional hyperresponsiveness of ISCLS-derived BOECs. Results from whole-genome sequencing done for 55 patients revealed previously unreported or extremely rare SNPs within the coding regions of several relevant genes including *NOS3* itself (encoding eNOS), *ATP2B2* (39), and *PPP2R1B*, several of which were predicted to be deleterious Combined Annotation Dependent Depletion ((CADD) score >10) (Supplemental Table 2). Because total eNOS expression was similar in ISCLS-derived BOECs and controls, whereas p-eNOS was increased even in quiescent cells, we focused further attention on candidate phosphatases. PP2A has a central function in the dephosphorylation of eNOS on Ser^1177^ (40), and our previous results from RNA-Seq performed on a smaller subset of BOECs suggested decreased expression of PP2A-encoding genes including *PPP2R1B*, *PPP2R3A*, and *PPP2R5A* in ISCLS cells compared with control cells (8). PP2A is a Ser/Thr phosphatase consisting of core catalytic (C), scaffold (A), and variable regulatory (B) subunits, and the structural subunits are required for full activity of the enzyme (41). We observed significantly lower PP2A-Aβ (encoded by *PPP2R1B*) protein expression in ISCLS-derived BOECs than in controls, whereas expression of PP2A-B and PP2A-C subunits was similar (Figure 8, C and D). By contrast, *PPP2R1B* mRNA expression did not differ between control and ISCLS-derived BOECs, suggesting that the aberrant PP2A-Aβ expression resulted from posttranscriptional mechanisms (Figure 8E). Overexpression of FLAG-PP2A-Aβ in ISCLS-derived BOECs significantly reduced VEGF-induced barrier disruption (Figure 8, F–H) and eNOS phosphorylation (Figure 8, I and J). These results suggest that reduced PP2A-A expression contributed to the hyperresponsiveness of ISCLS ECs to VEGF.

## Discussion

ISCLS is both rare and cryptogenic. Because the acute presentation resembles several more common conditions (e.g., sepsis), and unique biomarkers or diagnostic genetic assays do not yet exist, the diagnosis is frequently missed and/or delayed, with devastating consequences (1). Even in patients with an established diagnosis, clinicians are unable to determine when ISCLS will flare in each patient or predict how severe a given flare will be. The frequency of ISCLS attacks varies widely, and effective interventions for acute episodes are nonexistent. By examining vascular leakage in patients in situ, the molecular and biophysical behavior of cultured ECs in vitro, and a mouse model of disease, we discovered that an autonomous functional defect within the endothelium characterized by eNOS-dependent cytokine hypersensitivity may contribute to acute ISCLS crises.

Patients with ISCLS are typically asymptomatic between episodes and have normal physical examination findings. However, although we found no overt structural anomalies in the skin microvasculature in asymptomatic patients, VE-cadherin expression was reduced, suggesting the presence of ongoing, yet subclinical, endothelial dysfunction. This phenotype was partially recapitulated in ISCLS-derived BOECs, which displayed normal growth and morphology but had markedly exaggerated loss of intercellular adhesion when challenged with VEGF or histamine. Thus, the ISCLS endothelium may be primed for excessive leakage in the context of inflammation. Our findings also argue against a prominent role of EC injury or death in ISCLS-associated vascular leakage. We have not detected increased circulating levels of endothelial injury markers in acute ISCLS plasma (8), nor have we observed tissue hemorrhage in patients like that which can occur in infections with the Ebola/Marburg family of filoviruses due to direct endothelial cytotoxicity (42).

That the functional endothelial defect persists after passaging further suggests that the susceptibility of ISCLS-derived ECs to cytokine hypersensitivity is durable and raises the possibility of a genetic abnormality underlying the stress-induced endothelial phenotype. Although related studies of more common conditions have proposed that genetically determined variance in the host vascular response may contribute to the risk for other leakage pathologies (e.g., sepsis) (43–45), to our knowledge, there is no known hyperpermeability disorder in which the vasculature is genetically (or epigenetically) programmed to “hyperrespond” to otherwise routine stimuli.

One possible exception was reported about a boy with recurrent episodes of vascular leakage resembling ISCLS (1 fatal) associated with a monoallelic loss-of-function (LOF) mutation (D762V) in *ARHGAP5*, which encodes a GTPase activating protein for RhoB (p190BRhoGAP) that is expressed in ECs (46). However, it is unlikely that this child had ISCLS disease, considering that the clinical presentation was atypical (cerebral and pulmonary edema and hemorrhage, lack of peripheral edema or MGUS). Although dermal ECs isolated from this child postmortem recovered more slowly to TNF-α–induced barrier disruption, they had normal responses to histamine, unlike BOECs derived from adults with ISCLS. Moreover, this allele was not detected in our NIH ISCLS cohort, nor did we consistently detect other previously unreported or ultrarare LOF variants in *ARHGAP5*.

In fact, and consistent with the absence of familial inheritance of ISCLS, whole-exome sequencing performed on leukocyte DNA samples from 7 pediatric probands with classic ISCLS, their immediate relatives, and 9 unrelated adults failed to identify any de novo, previously unreported (or even highly rare) single-nucleotide variants shared among any 2 patients (47). Ultra-rare mutations in noncoding DNA or mosaicism could nonetheless contribute to the pathogenesis of ISCLS. Simultaneous whole-genome sequencing of EC lines and unrelated tissues may be needed to detect low-frequency somatic variants confined to the endothelium.

Our mechanistic studies point to an important contribution of eNOS dysregulation in the pathogenesis of ISCLS. eNOS promotes endothelial barrier dysfunction through several mechanisms including enhancement of Src-dependent phosphorylation of VE-cadherin on Tyr^685^ in response to VEGF (48). Consistent with this mechanism, we detected increased VE-cadherin (p-Tyr^685^) in histamine-challenged ISCLS skin vasculature compared with controls. The aggregation of p-eNOS at the PM of ISCLS BOECs could signify an underlying commitment to leakage and increased sensitivity to agonists. Published studies of subcellular localization in human umbilical vein ECs (HUVECs) demonstrate that eNOS is acylated and localizes predominantly at the PM within caveolae and on the cytoplasmic side of the Golgi membrane (28). Studies of *Nos3^–/–^* ECs reconstituted with differentially localized eNOS mutants have further suggested that PM-targeted eNOS is constitutively phosphorylated on Ser^1177^ and produces more NO than a Golgi-targeted mutant in response to Ca^2+^- or Akt-activating stimuli (49). Since caveolin-1 is a negative regulator of eNOS activity (50), it may be informative to determine whether these eNOS aggregates are excluded from PM-associated caveolin-rich microdomains in ISCLS-derived BOECs.

The significance of increased AMPK phosphorylation in ISCLS BOECs is unclear. Our results are in line with those from earlier studies of HUVEC-derived and lung ECs from *Ampkα1^–/–^* mice, which have intact VEGF-induced eNOS phosphorylation (33). However, more recent studies of brain microvascular cells and mouse retinae ex vivo have pointed to an indispensable role for AMPK in VEGF- and bradykinin-evoked permeability downstream of Ca^2+^ and CAMKKβ (34). The function of AMPK in permeability may thus vary with endothelial heterogeneity. Increased AMPK activity in quiescent ISCLS ECs also raises the specter of a metabolic phenotype reflective of an increased intracellular AMP/ATP ratio; further examination of ISCLS BOEC metabolism in the presence or absence of inflammatory stimuli may be warranted.

Last, we detected reduced PP2A-A expression in ISCLS BOECs, which may in turn have augmented and/or prolonged eNOS activity. PP2A-A knockdown by RNAi destabilizes the expression of B and C subunits and thereby compromises PP2A enzymatic activity (51). Somatic mutations in *PPP2R1A* and *PPP2R1B* are frequently detected in cancer, and functional haploinsufficiency promotes tumorigenesis (52). We did not detect any well-known cancer-associated variants in PP2A-A– or PP2A-B–encoding genes in ISCLS, and thus further studies are required to determine the significance of *PP2R1A-B* mutations for PP2A-A expression in ISCLS.

We believe our discovery of endothelial hypersensitivity in ISCLS has significant implications for the treatment of acute attacks. Although ISCLS flares are frequently triggered by viral infections and accompanied by transient increases in circulating proinflammatory and angiogenic cytokines (e.g., TNF-α, CXCL10, CCL2, IL-6, VEGF, angiopoietin 2), the cytokine storm may have already peaked by the time the patient presents with hypotensive shock (21, 26). Consequently, inhibition of specific humoral factors or their receptors (histamine, VEGF, bradykinin) or more broadly active antiinflammatory agents (e.g., corticosteroids, immunosuppressives) confers no benefit in acute ISCLS crises (2, 53, 54). However, administration of methylene blue, a NO scavenger, was reported to reverse hypotension in a single patient (55). Further detailed characterization of the eNOS-dependent and -independent endothelial defect(s) in ISCLS-derived ECs may uncover new and more effective therapeutic targets.

## Methods

### Sex as a biological variable.

The prevalence of ISCLS is similar in men and women; therefore, we did not consider sex as a biological variable. All available patient-derived cell lines, regardless of sex, were used. Approximately equal numbers of male and female mice were used for functional studies.

### Reagents, chemicals, and antibodies.

Collagen I, l-NAME, l-NAME–cysteine, gelatin (from porcine skin), thrombin (from human plasma), histamine, EB, STO-609, probenecid, and DAPI were all purchased from MilliporeSigma. Recombinant human VEGF165 was obtained from PeproTech. Lipofectamine and phalloidin were purchased from Thermo Fisher Scientific. Complete and PhoSTOP inhibitor tablets were obtained from Roche. A complete list of the antibodies used is provided in Supplemental Table 3.

### Isolation and expansion of BOECs.

PBMCs were isolated from whole blood and cultured in Endothelial Growth Medium 2 (EGM-2) (Lonza) on collagen I–coated dishes as previously described (11). The culture medium was changed every other day for several weeks until discrete colonies formed, and BOECs were expanded according to previously published protocols (25). BOECs from anonymized healthy donors were used as controls for all studies, and cells were used up to 20 passages.

### RNA interference.

BOECs were transfected with the ON-TARGETplus SMARTpool siRNA for human *NOS3* (L-L-006490-00-0005) or with a control nontargeting siRNA (D-001810-10-20) (final concentration of 12.5 nM) using Dharmafect Transfection Reagent 4 (Dharmacon). Expression and functional studies were done 72 hours after transfection.

### Lentiviral transfection.

Vector for the negative control virus (EX-NEG-Lv203) and virus preparation reagents were obtained from GeneCopoeia. Virus was prepared according to the manufacturer’s instructions. Briefly, 2.5 μg DNA-EndoFectin complex (Lenti-Pac HIV expression packing kit, catalog LT001), diluted in Opti-MEM I medium (Gibco, Thermo Fisher Scientific, catalog 31985062), was transfected into HEK293A cells. The next day, transfection medium was replaced with fresh DMEM and 1:500 (v/v) Titer Boost Reagent (GeneCopoeia). Forty-eight hours after transfection, pseudovirus-containing medium was harvested and concentrated with Lenti-X concentration solution (Takara) overnight at 4°C (1:3, v/v). The virus pellet was resuspended in complete BOEC medium and stored in aliquots at –80°C. BOECs were infected with FLAG-PPP2R1B virus (GeneCopoeia, catalog EX-W0293-Lv203) or with the negative control virus at confluence (1:120, v/v) in the presence of polybrene (8 μg/mL, TR-1003-G, MilliporeSigma) for 2 hours at 4°C and then transferred to 37°C. Cells were evaluated 20–24 hours after infection.

### Immunoblotting and immunofluorescence.

Cell lysates were prepared in radioimmunoprecipitation buffer containing protease and phosphatase inhibitor cocktails and electrophoresed on NuPAGE gels (Thermo Fisher Scientific) before transfer onto nitrocellulose or PVDF membranes. After immunoblotting, blots were detected with near-infrared–conjugated secondary antibodies using the Li-COR Odyssey 3000 imager (LI-COR Biosciences). Signals were quantified using ImageStudio (LI-COR) or ImageJ software (NIH). For immunofluorescence, BOECs were grown to confluence on collagen I–coated Chamberwell slides (Nunc) and serum starved with 0.2% BSA in EBM-2 medium (5 hours, 37°C). Following stimulation, cells were fixed in 4% paraformaldehyde in PBS (15 minutes, room temperature [RT]) and permeabilized with 0.2% Triton X-100 in PBS (5 minutes, RT). After incubation in blocking buffer (3% BSA/0.1% Tween-20/5% goat serum in PBS, 1 hour, RT), cells were incubated with primary antibodies in blocking buffer overnight at 4°C. Cells were washed 4 times with wash buffer (0.1% Tween-20 in PBS), followed by incubation with goat anti–mouse or anti–rabbit fluorophore-conjugated secondary antibodies in blocking buffer. Cells were then incubated with DAPI (1 μg/mL, Invitrogen, Thermo Fisher Scientific) for 5 minutes at RT and mounted onto glass coverslips using ProLong antifade mounting medium (Invitrogen). Skin sections were fixed in cold methanol and then stained and blocked with Image-iT FX signal enhancer (Invitrogen, Thermo Fisher Scientific) prior to incubation with antibodies and processing as above. Images were acquired at ×63 magnification using a Leica DMI8 Sp8 confocal microscope.

For quantification of 3D-rendered images of skin biopsies and eNOS immunostaining in BOECs (Figure 1 and Supplemental Videos 1 and 2), Imaris software was used for 3D rendering and volumetric measurement. Multiposition tiling was used to obtain a larger or entire area of skin to observe a global view of the spatial distribution. For 1D images (Figure 2), Imaris and ImageJ were used to calculate the absolute fluorescence intensity and ratiometric analysis (percentage of area), respectively. In experiments using cell monolayers (Figure 4), areas of VE-cadherin disruption on cell membranes were identified using ImageJ in more than 5 separate fields/condition for each experiment. The “auto-threshold” function was used to first limit analysis of 8-bit images to positively stained areas. Numerical values were obtained for the total cell perimeter, and all linear regions of the membrane lacking positive signal were identified manually using the “freehand” tool. The final values (percentage of membrane gaps) were calculated as the sum of nonstained areas divided by the cell perimeter.

### Ca^2+^ measurements.

BOECs were plated in 96-well, black-walled plates (1 × 10^5^ cells/well). Ca^2+^ Fluo-6 indicator (FLIPR Calcium 6 Assay Kit, Molecular Devices) and probenecid (1 mM) were added to each well containing serum-free EGM-2 for 2 hours. Agonists were added robotically to wells using the FlexStation III instrument (Molecular Devices), and fluorescence was measured every 1.5 seconds for 180 seconds. Each reading was divided by the initial value to obtain the normalized Ca^2+^ value.

### Phosphoproteome profiling.

A human Phospho-Kinase Array Kit was purchased from R&D Systems, and analysis was performed according to the manufacturer’s instructions. Briefly, BOECs were serum starved in EGM-2 for 5 hours prior to stimulation with VEGF (100 ng/mL) for 15 minutes. Cell lysates were prepared as described above and incubated with membranes overnight at 4^o^C. Signals were detected and quantified as outlined for immunoblots.

### ECIS.

BOECs were plated on gelatin-coated wells (4 × 10^4^ cells/well) containing gold-plated electrodes (8W10E+ PET arrays, Applied BioPhysics). Electrodes were cleaned with cysteine (100 mM) overnight prior to coating. Cells were incubated overnight in EGM-2 followed by serum starvation in Endothelial Basal Medium (Lonza) containing 0.2% BSA for 5 hours at 37°C prior to stimulation. TER was recorded over a period of 20 hours. Each condition was measured in duplicate in a single experiment and then averaged. Absolute resistance values were normalized by subtracting the resistance at time zero (before treatment); the maximal change in resistance was calculated as the percentage of change over time zero.

### Mouse ISCLS model.

Aged (>6 months of age) SJL/J mice (The Jackson Laboratory) were used to assess vascular leakage as previously described (10) and as outlined in Figure 6, A and F. Briefly, mice were injected with 100 μL of 2% EB in PBS retro-orbitally. Immediately thereafter, mice were injected i.p. with 100 μL histamine in PBS (2.5 mg/kg BW). Fifteen minutes after injection, mice were deeply anesthetized by isoflurane inhalation and perfused with 5 mL heparinized PBS through the left ventricle to remove residual intravascular EB. Tissues were harvested and heated at 95°C for 1 hour to obtain tissue dry weights. A Miles assay was performed to assess VEGF-induced vascular leakage in skin. Briefly, mice were injected i.p. with pyrilamine maleate (4 mg/kg BW, MilliporeSigma) 30 minutes prior to injection with EB dye to reduce background permeability during handling. Mice were then injected with EB via retro-orbital injection, as before, followed by intradermal injections of VEGF or saline (50 μL total volume). Thirty minutes after the intradermal injection, the dorsal skin was collected with a 12 mm biopsy punch. EB was extracted from dried tissues with formamide (MilliporeSigma; 56°C for 48 hours). The amount of EB in each sample was determined by measuring the absorbance at 620 nm, and results are expressed as the EB dye amount (ng) per 100 mm^2^ of skin or tissue weight (mg), with quantification against a standard curve.

### Whole-genome sequencing.

Variants were called jointly using the publicly available genome-seek pipeline (https://github.com/OpenOmics/genome-seek). Briefly, raw fastq files were trimmed using fastp (56) and aligned with bwa-mem2 to the GRCh38 human genome reference. Samblaster version 0.1.26 (57) was used to flag PCR duplicates, and BAM files were sorted using samtools, version 1.16.1 (58). Final BAM files were used as input to DeepVariant version 1.4.0 (59) to generate genomic variaant call files (gVCFs), and GLNexus version 1.4.1 (60) was used to joint-genotype the cohort. Variants were annotated with gnomAD (61) allele frequency and the CADD (62) score using open-cravat version 2.2.5 (63). Additional annotation tools were used, including Loss/Gain Function Prediction software (https://itanlab.shinyapps.io/goflof/) (64) to predict variant functional effect.

### Statistics.

All statistical analyses were performed using GraphPad Prism (GraphPad Software). An unpaired, 2-tailed Student’s *t* test was used to compared 2 groups, and a nonparametric Mann-Whitney *U* test was used for non-normally distributed data. A χ^2^ test was used to compare demographic contingency variables. A 1-sample Student’s *t* test was used to compare differences between 1 sample and a normalized control (for example, to 100%). A 1- or 2-way ANOVA was used for analysis of multiple groups, with the post hoc multiple-comparison tests recommended by Prism. Pearson’s coefficients were calculated to assess correlations between parameters. A *P* value of less than 0.05 was considered statistically significant.

### Study approval.

After providing written informed consent, patients with ISCLS were enrolled in a clinical study protocol approved by the IRB of the NIH (09-I-0184). Mice were housed and bred at an American Association for the Accreditation of Laboratory Animal Care–accredited (AAALAC-accredited) facility at the NIH. The animal study proposal (LAD3E) was approved by the IACUC of the NIAID, NIH.

### Data availability.

Values for all data points in graphs are reported in the Supporting Data Values file. Whole-genome sequencing data have been deposited in the Database of Genotypes and Phenotypes (dbGaP) (accession no. phs003261.v1.p1).

## Author contributions

AJA, WSC, ZX, and AD performed experiments and analyzed and interpreted data. AZD provided cell lines and edited the manuscript. ARE and LAS recruited and cared for patients, performed skin testing and biopsies, and edited the manuscript. SMP analyzed and interpreted data and edited the manuscript. KMD supervised the project, performed experiments, analyzed and interpreted data, and wrote the manuscript. SP and JBL analyzed and interpreted genomic sequencing data. All authors read and approved the final manuscript.

## Supplementary Material

Supplemental data

Unedited blot and gel images

Supplemental video 1

Supplemental video 2

Supporting data values

## Version 1 03/19/2024

In-Press Preview

## Version 2 05/15/2024

Electronic publication
