# Supplementary material for: Intrinsic endothelial hyperresponsiveness to inflammatory mediators drives acute episodes in models of Clarkson disease
Source: J Clin Invest. 2024 Mar 19;134(10):e169137. doi: 10.1172/JCI169137 (PMC11093607; doi:10.1172/JCI169137)
Supplement: Supplemental data [file jci-134-169137-s151.pdf]

Supplemental Data

Supplemental Figures 1-8

Supplemental Tables 1-3

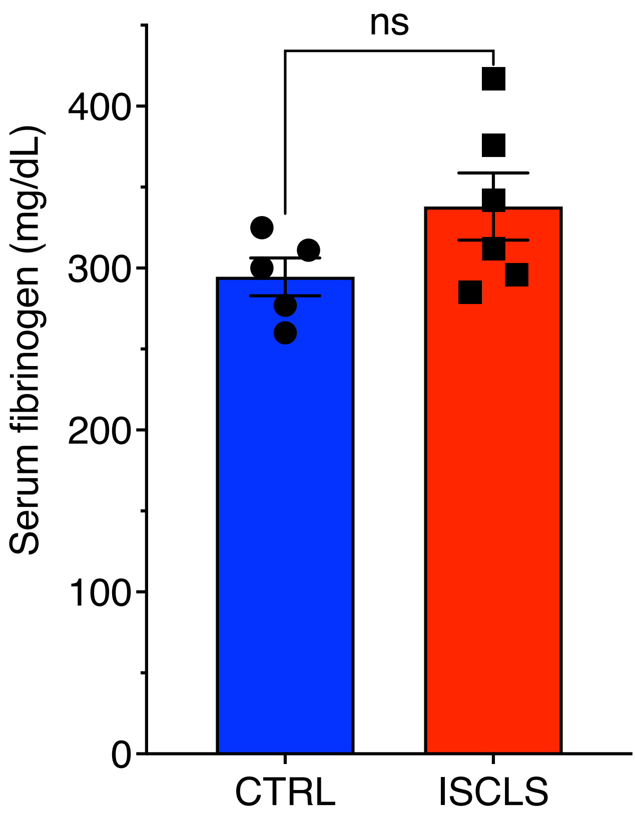

Supplemental Figure 1 Serum fibrinogen levels. Serum fibrinogen levels in asymptomatic subjects; mean ± s.e.m. of n=6 ISCLS, 5 CTRL, ns, unpaired *t* test. Normal range=177-477 mg/dL.

Fig S2

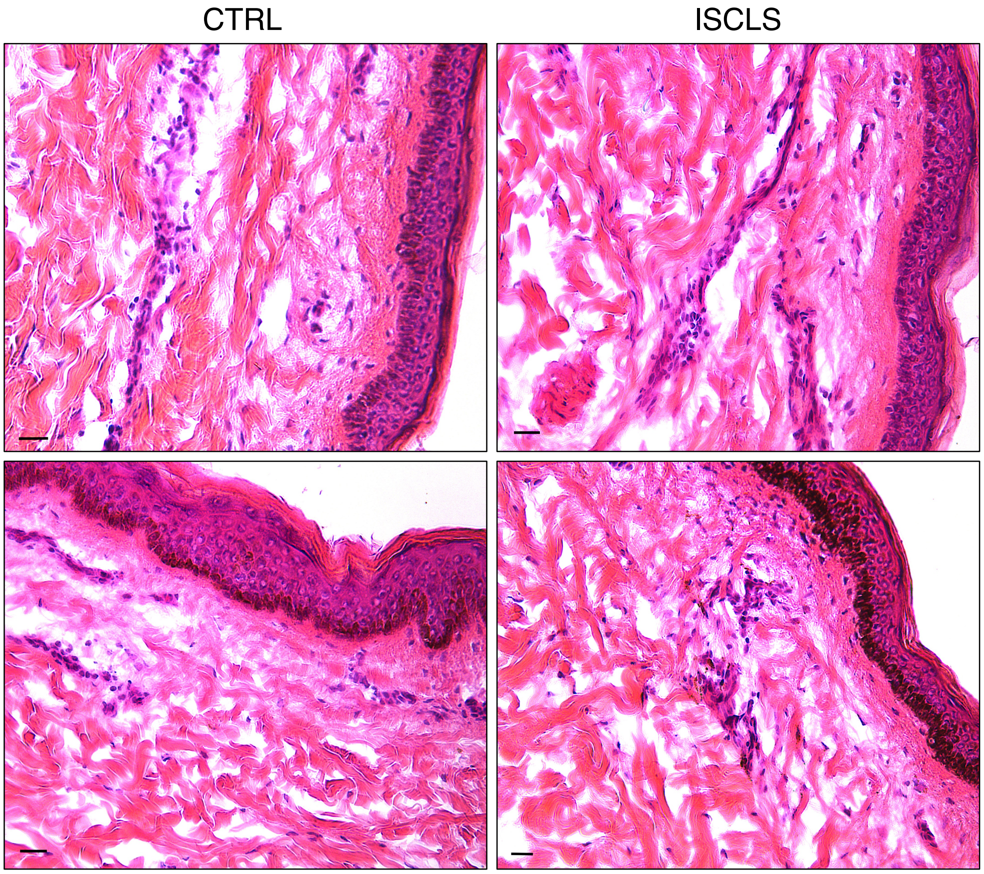

Supplemental Figure 2 Skin biopsies in asymptomatic subjects. Skin biopsies of saline-challenged skin in patients with ISCLS or controls (n=2/group) stained with hematoxylin and eosin. Scale bar=25 mm; original magnification 63x.

Fig S3

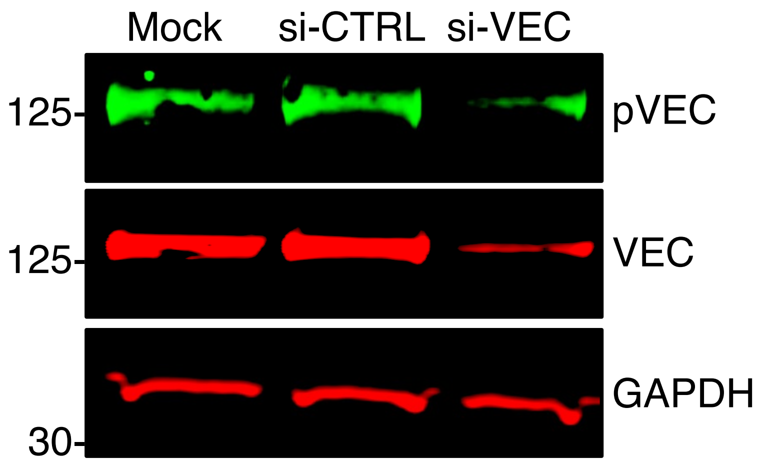

Supplemental Figure 3 Specificity of phospho-VE-cadherin antibody. HMVECs were left untreated (“mock”) or transfected with non-targeting control or *CDH5*-specific (“VEC”) siRNAs. 48 hours after transfection, cells were stimulated with VEGF (100 ng/mL for 15 minutes). Cell lysates were immunoblotted with VE-cadherin, phospho-VE-cadherin, and GAPDH antibodies. Images represent 2 similar experiments.

Fig S4

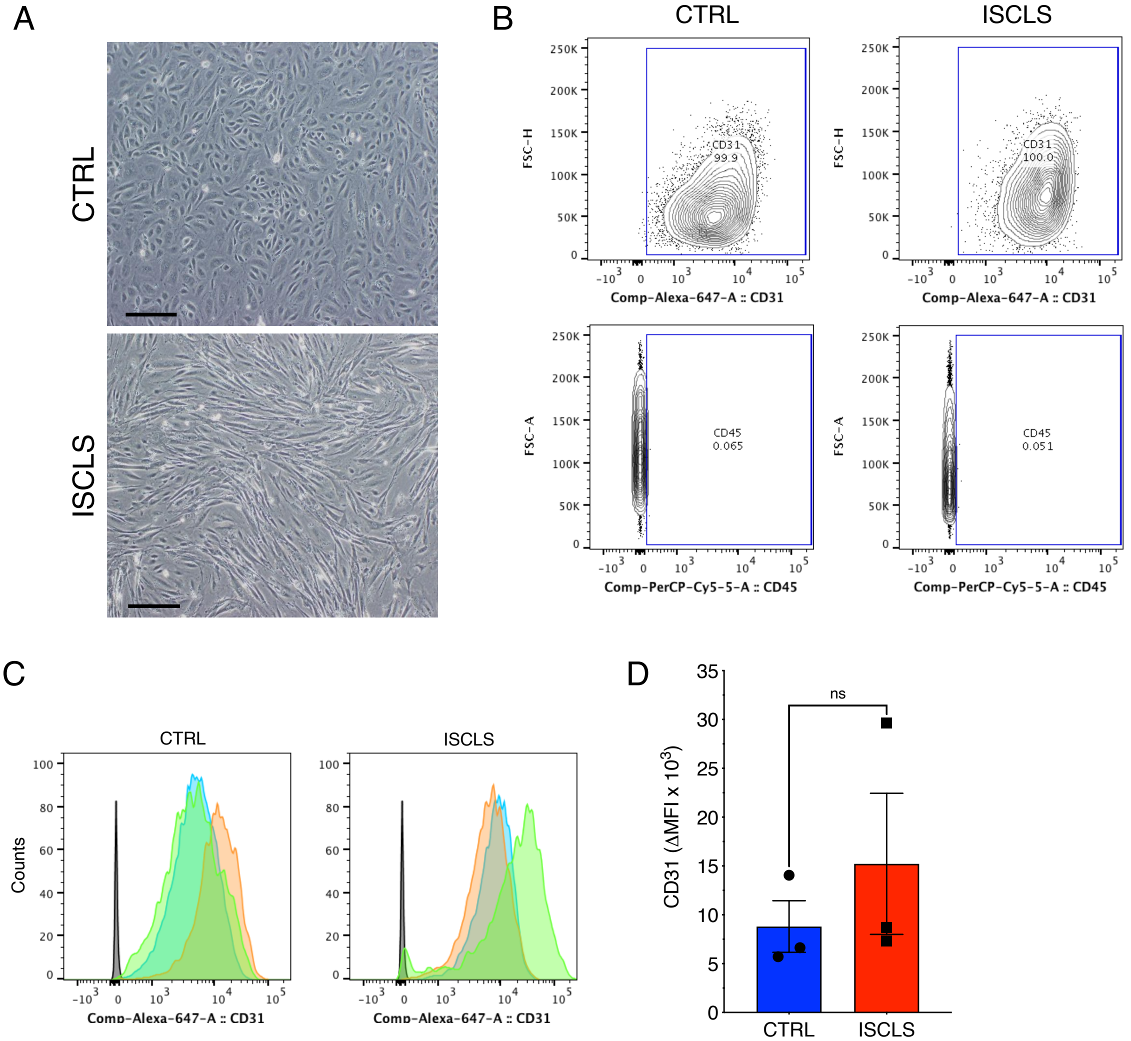

Supplemental Figure 4 Characterization of BOECs. (A) Brightfield images of control or ISCLS-derived BOECs. Scale bar=200 mm. (B-C) Dot plots of CD31 and CD45 immunostaining (B); representative histograms of CD31 staining (C). (D) Quantification of geometric mean fluorescence intensity (GMFI) determined by flow cytometry; mean ± s.e.m. of n=3 donors/group.

Fig S5

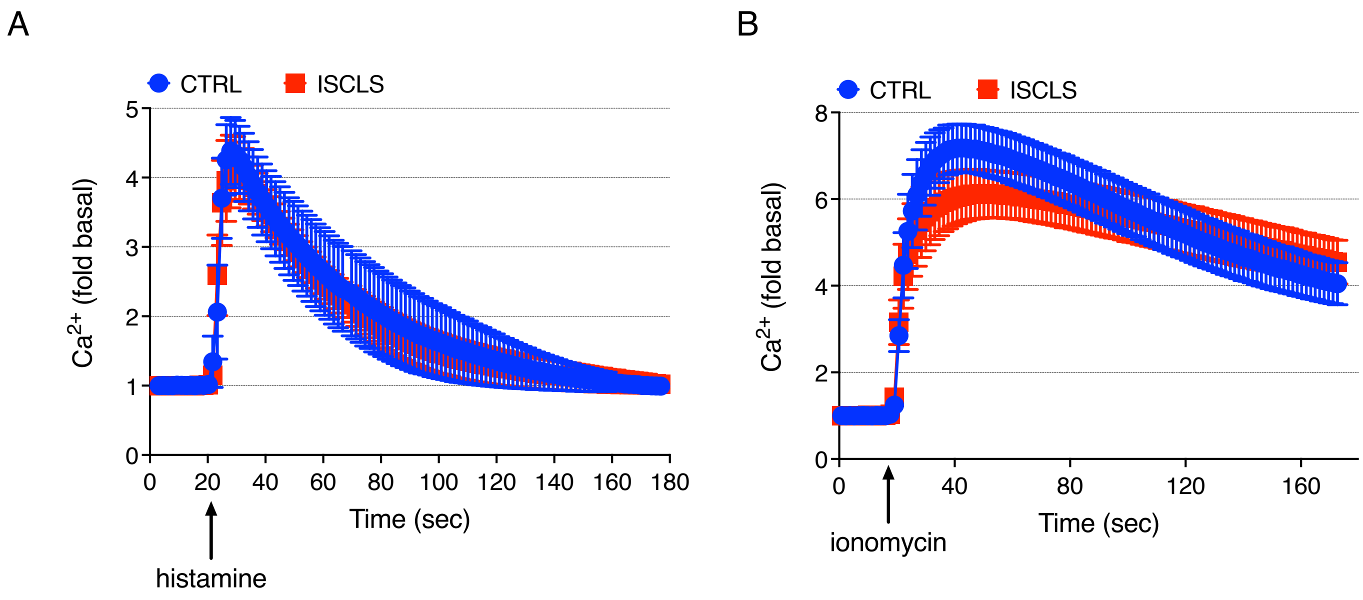

Supplemental Figure 5 Ca^{2+} flux in ISCLS-derived ECs. ((A-B) Relative intracellular Ca^{2+} concentrations in CTRL (blue) or ISCLS (red) BOECs stimulated with histamine (0.8-2 mM, A) or ionomycin (1-2 mM, B); mean ± s.e.m. of n=3 donors/group analyzed in 4-5 independent experiments. Arrows indicate time of agonist addition.

Fig S6

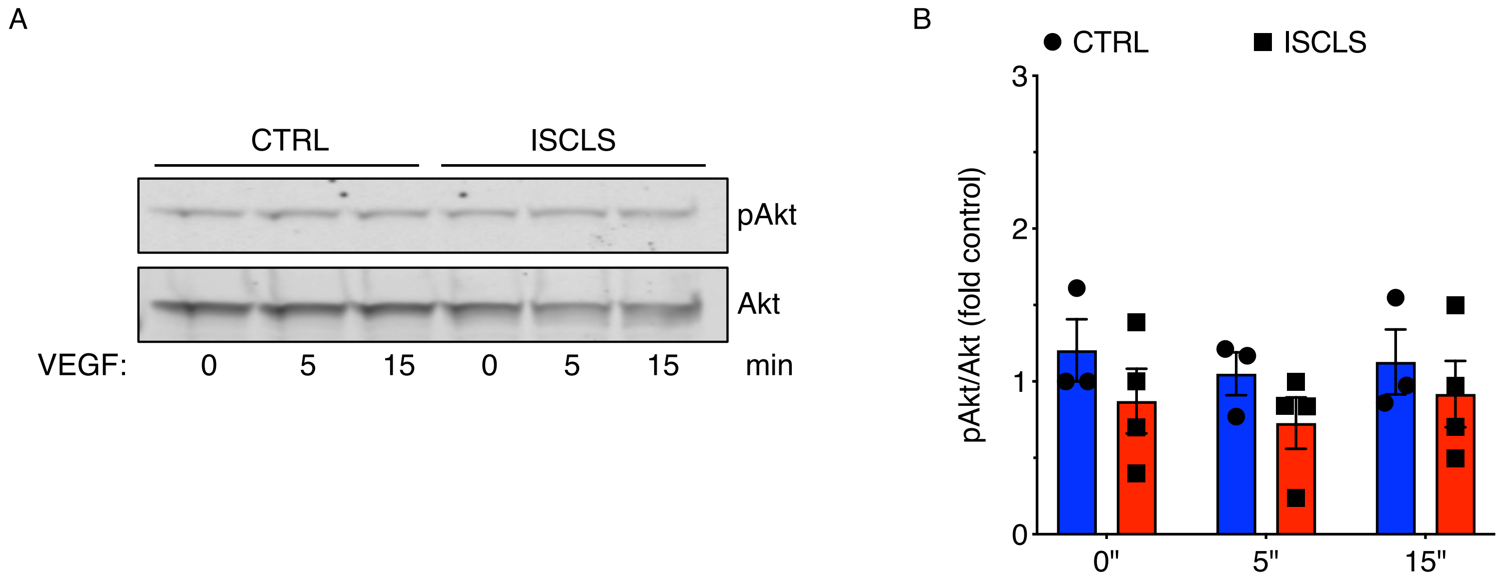

Supplemental Figure 6 Akt phosphorylation in BOECs. (**A-B**) Representative blot (A) and quantification of phospho-Akt and total Akt (B) in BOECs stimulated with VEGF (100 ng/mL) for the indicated times. Results are compiled from n=3 CTRL and n=4 ISCLS-derived BOECs.

Fig S7

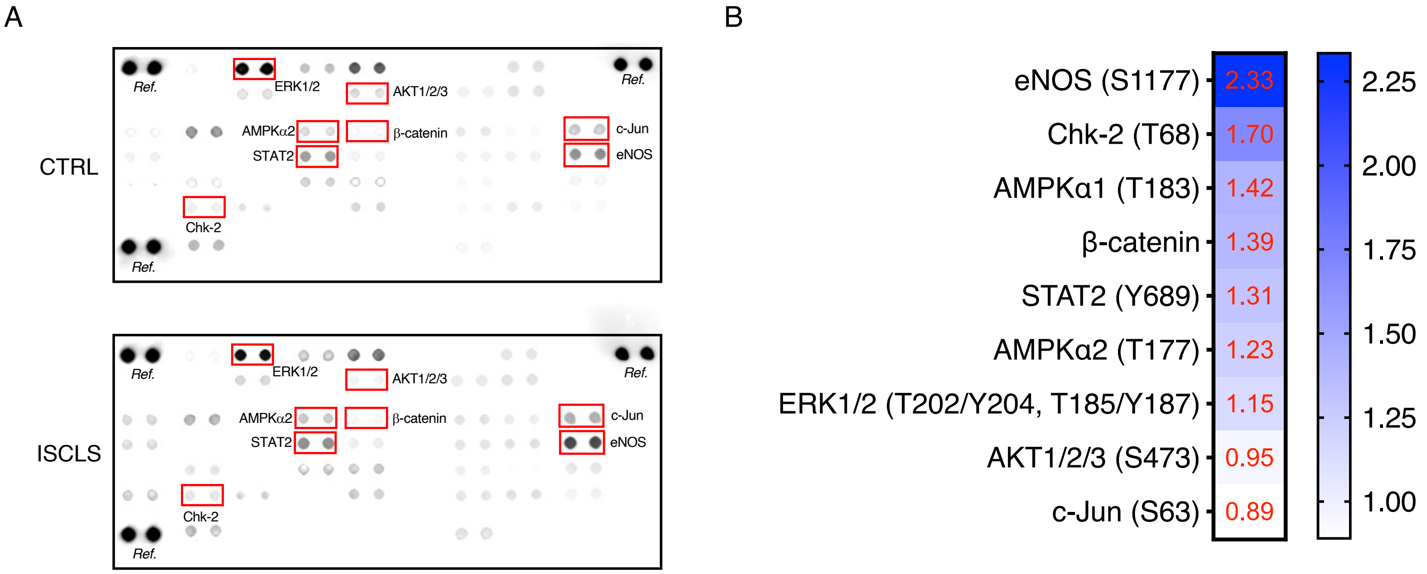

Supplemental Figure 7 Phosphoproteomic analysis of VEGF-treated BOECs. (A) Representative Proteome Profiler^{TM} blots incubated with lysates from BOECs stimulated with VEGF (100 ng/mL x 15 minutes). (B) Heat map of top differentially phosphorylated proteins in ISCLS vs. CTRL BOECs; mean of n= 2 CTRL, 2 ISCLS).

Fig S8

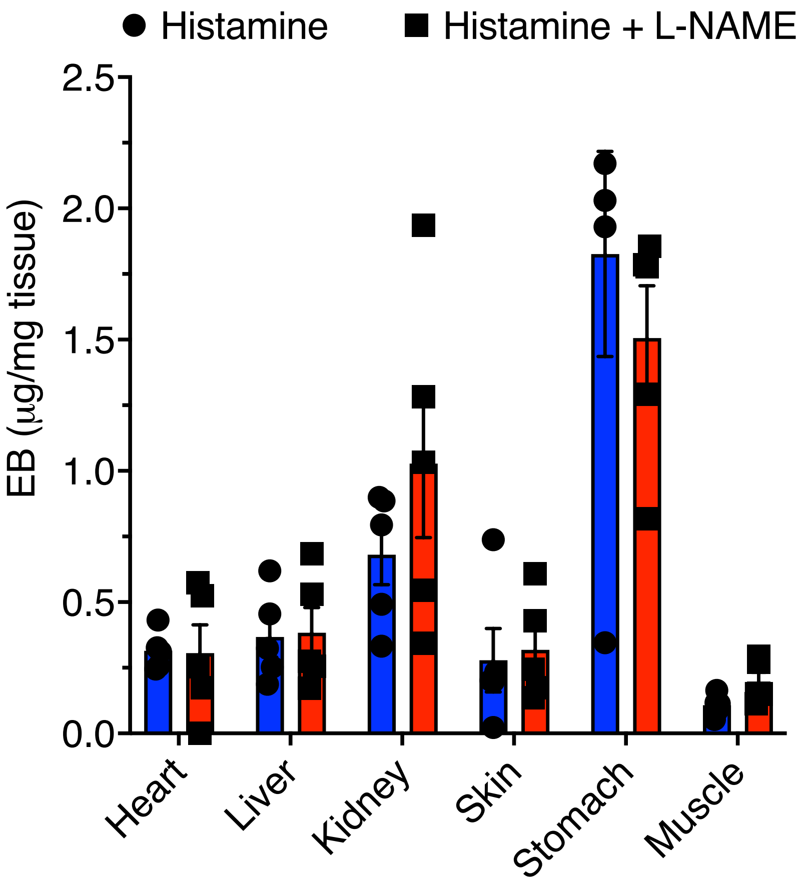

Supplemental Figure 8 Effect of L-NAME on histamine-induced vascular leakage in histamine-resistant mice. C57BL/6 mice were pretreated with vehicle or L-NAME (10 mg/kg) followed by challenge with histamine (2.5 mg/kg) i.p. and analysis of extravasated Evans Blue (EB). Mean ± s.e.m. of n=5 mice/group.

Supplemental Table 1 Patient demographics

**Donors**	**CTRL (n=15)**	**ISCLS (n=21)**
Age (years)[#]	43 (20-61)	56 (25-75)
Sex (% female)	46.7	36.4
Ethnicity (%non-white)	40	18
MGUS (%)	0	86

[#]At time of blood/sample collection (median, range), ***p*=0.002, Mann-Whitney

Supplemental Table 2 Novel or ultra-rare high-impact SNPs in eNOS-related genes in ISCLS subjects

**CHR**	**POS**	**REF**	**ALT**	**Gene**	**SO**	**Gnomad3_AF**	**SampleAF**	**CADD**
chr3	10359892	C	T	ATP2B2	Missense Variant	0.00778579	0.018	22.2
chr3	10410710	C	CTCGTGTGGGAG	ATP2B2	Frameshift Elongation		0.045	
chr3	10410712	CCTCCCACACGA	C	ATP2B2	Frameshift Truncation		0.045	
chr11	111764842	C	T	PPP2R1B	Missense Variant	0.00700755	0.018	20.5
chr7	150995203	G	GC	NOS3	Frameshift Elongation	0.000006985	0.055	
chr7	150996481	G	GC	NOS3	Frameshift Elongation	1.44991E-05	0.045	
chr7	150998649	C	CG	NOS3	Frameshift Elongation		0.018	
chr7	150999023	T	G	NOS3	Missense Variant	0.755025	0.618	10.07
chr7	151001310	A	AG	NOS3	Frameshift Elongation		0.055	

Supplemental Table 3 Antibodies used

Antibody	Source	Species	Clone	Catalogue number	Fluorophore	Dilution	Application
CD31	Ebioscience	Mouse	JC70	14-0318-93	none	1:100	IF
CD31	Biolegend	Mouse		303126	AF594	1:100	IF
CD31	Biolegend	Mouse	WM59	302126	AF594	1:200	IF
CD31	BIoLegend	Mouse	WM59	303111	AF647	1:200	FC
Fibrinogen (human)	Abcam	Mouse		ab4217	FITC	1:300	IF
SMAA	Novus Biologicals	Mouse	1A4/asm-1	NBP2-34522AF488	AF488	1:100	IF
Collagen type IV	Abcam	Rabbit		ab6586	none	1:200	IF
VE-cadherin	BD Pharmingen	Mouse	55-7H1	561567	AF647	1:100	IF
VE-cadherin pTyr685	Millipore	Rabbit		ABT1760-AF488	AF488	1:75	IF
CD45	BIoLegend	Mouse	2D1	368503	PerCP-Cy5.5	1:200	FC
VEGFR2	R&D Systems	Goat		ABT1760-AF488	none	1:1000	WB
H1 receptor	Santa Cruz Biotechnology	Mouse	G-11	sc-374621	none	1:500	WB
eNOS	Cell Signaling Technology	Mouse	6H2	5880S	none	1:500	WB/IF
eNOS pSer1177	Cell Signaling Technology	Rabbit		9571S	none	1:500	WB
eNOS pSer1177	ThermoFisher	Rabbit		PA5-104858	none	1:500	WB
eNOS pSer1177	Abcam	Rabbit		ab184154	none	1:500	IF
Akt	Cell Signaling Technology	Mouse	40D4	2920S	none	1:1000	WB
Akt pSer473	Cell Signaling Technology	Rabbit	D9E	4060S	none	1:1000	WB
AMPK	Cell Signaling Technology	Mouse	F6	2793S	none	1:500	WB
FLAG	Sigma-Aldrich	Mouse	M2	F1804	none	1:1000	WB
AMPK pThr172	Cell Signaling Technology	Rabbit	40H9	2535S	none	1:2000	WB
PP2A-A beta	Abcam	Rabbit	EPR10158	ab154815	none	1:1000	WB
PP2A-A alpha	Cell Signaling Technology	Rabbit	81G5	2041S	none	1:1000	WB
PP2B-B	Cell Signaling Technology	Rabbit	100C1	2290S	none	1:1000	WB
PP2A-C	Cell Signaling Technology	Rabbit	52F8	2259S	none	1:1000	WB
Anti-mouse IgG	ThermoFisher	Donkey		A21203	AF594	1:300	IF
Anti-rabbit IgG	ThermoFisher	Donkey		A21206	AF488	1:300	IF
