# Supplementary material for: Intrinsic endothelial hyperresponsiveness to inflammatory mediators drives acute episodes in models of Clarkson disease
Source: J Clin Invest. 2024 Mar 19;134(10):e169137. doi: 10.1172/JCI169137 (PMC11093607; doi:10.1172/JCI169137)

Full unedited gels for Supplemental Figure 3

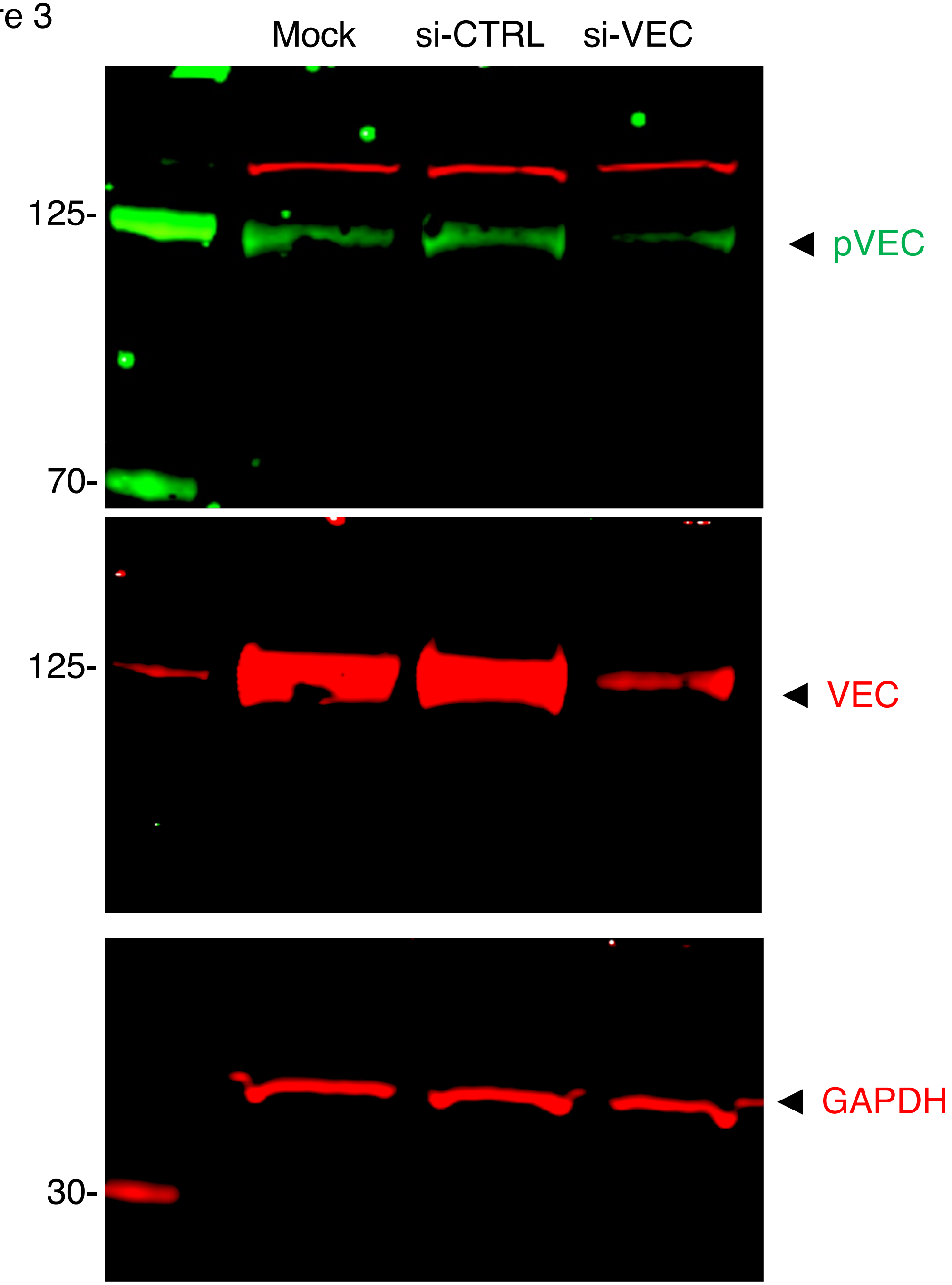

Full unedited gels for Figure 5A

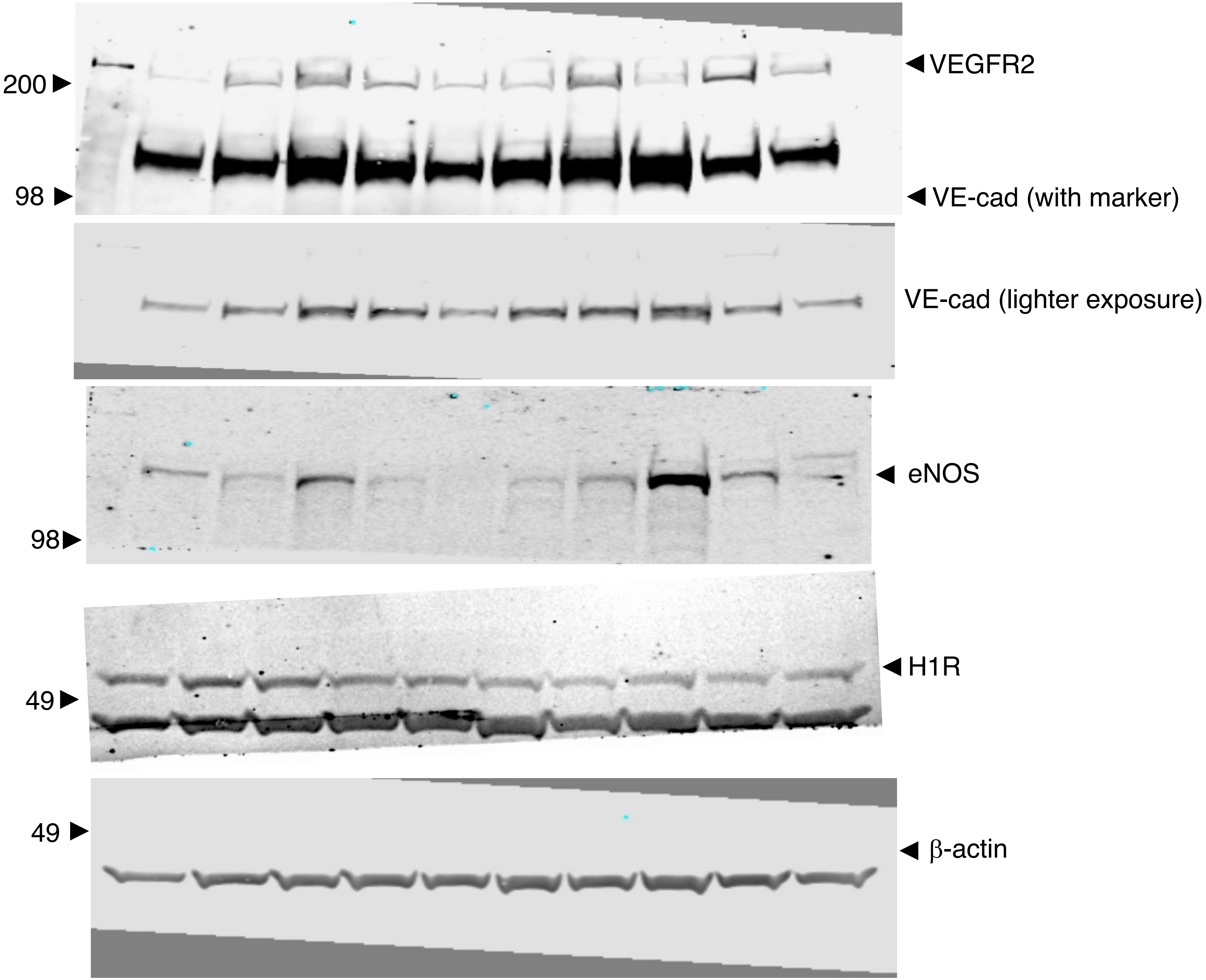

Full unedited gels for Figure 5D

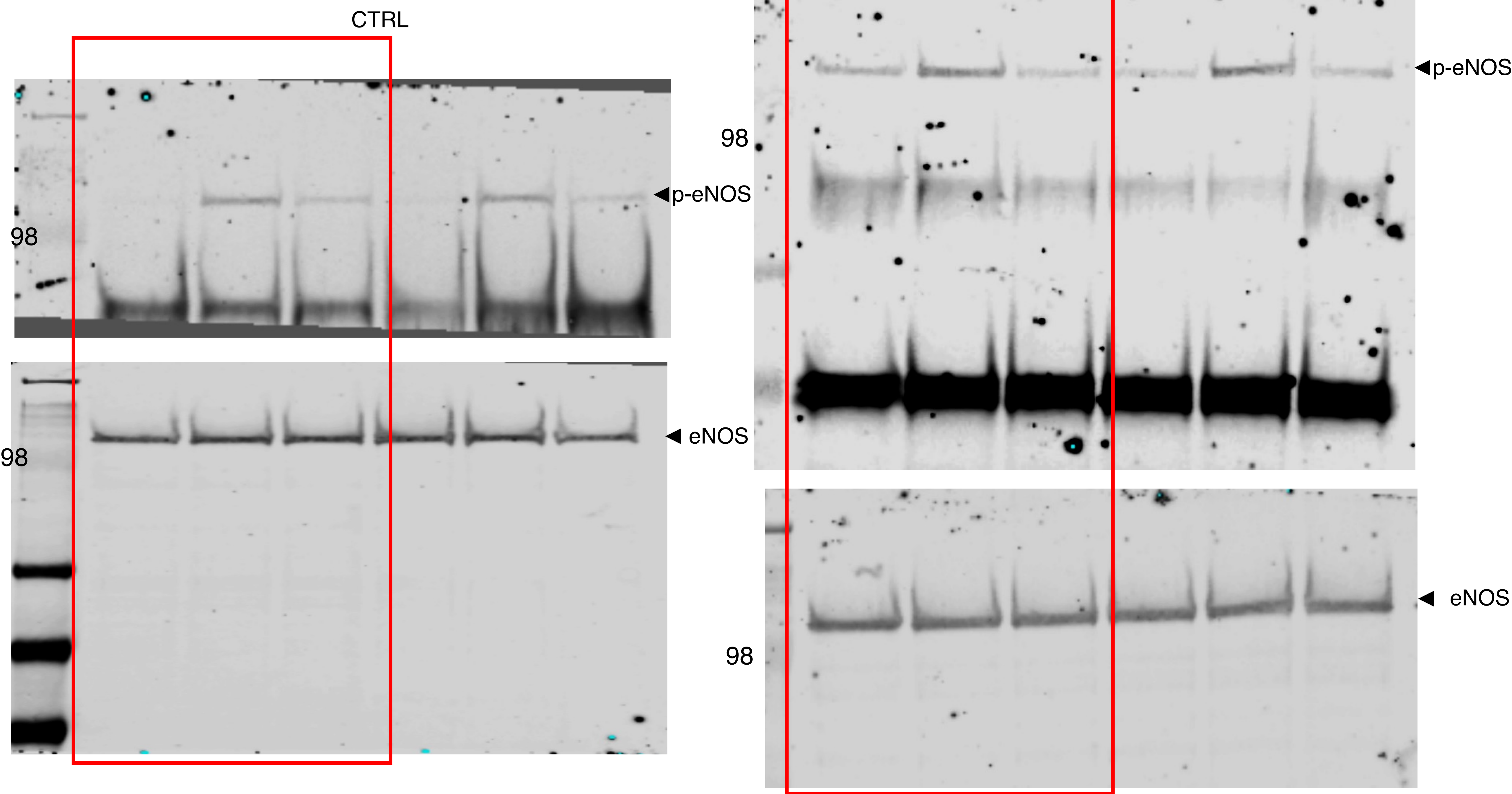

Full unedited gels for Figure 5F

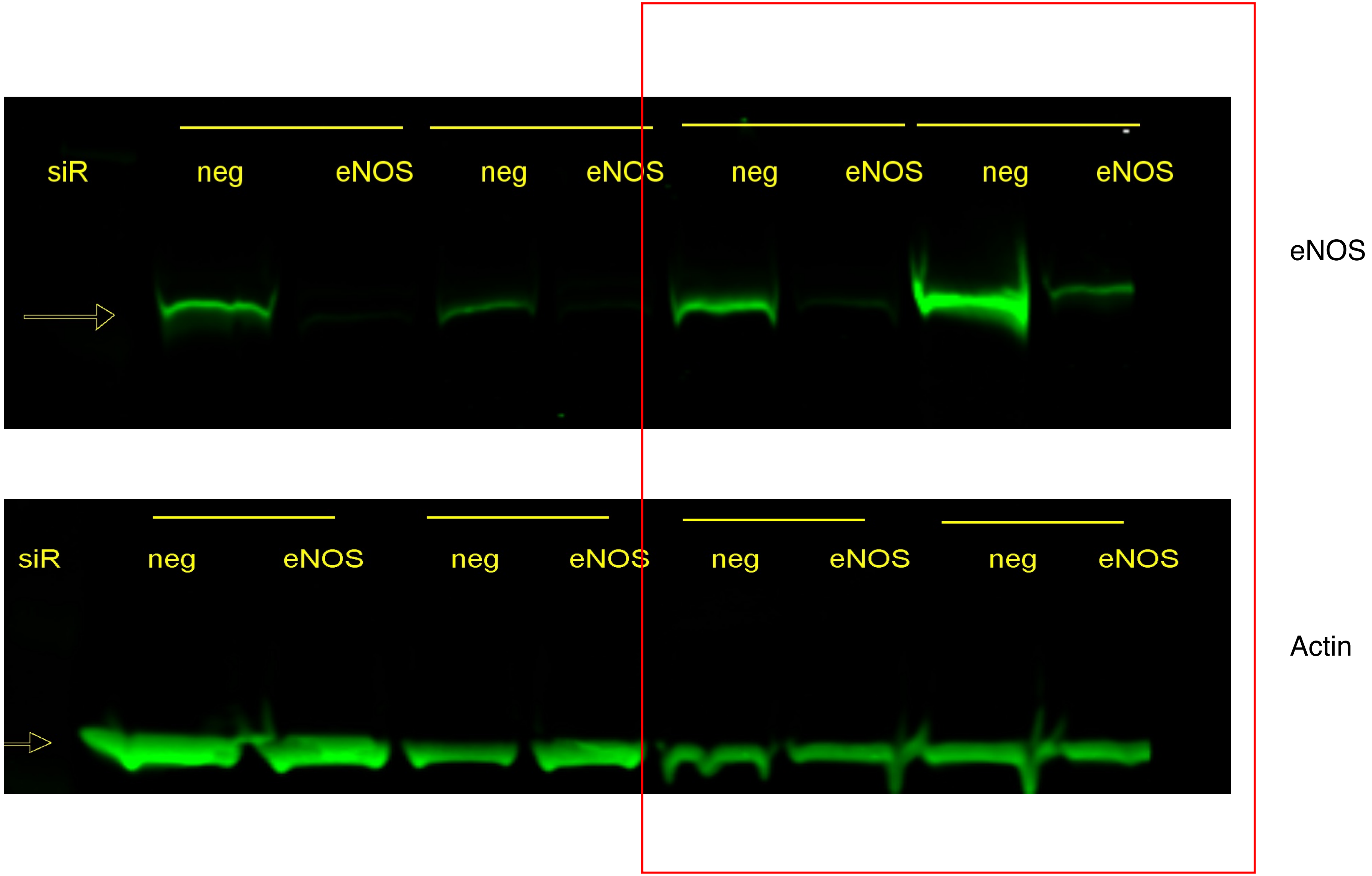

Full unedited gels for Figure 7A

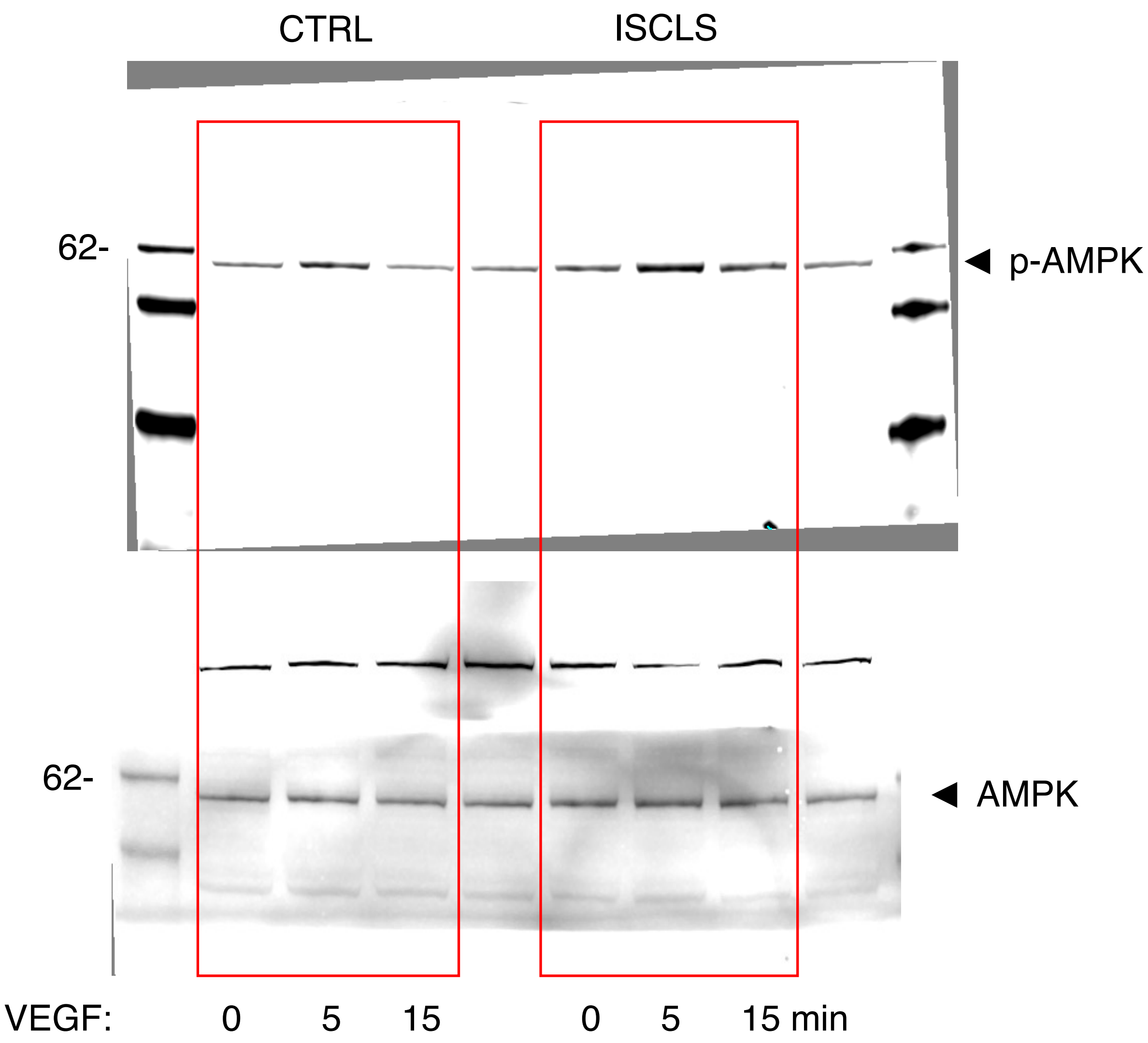

Full unedited gels for Figure 7C,G

C

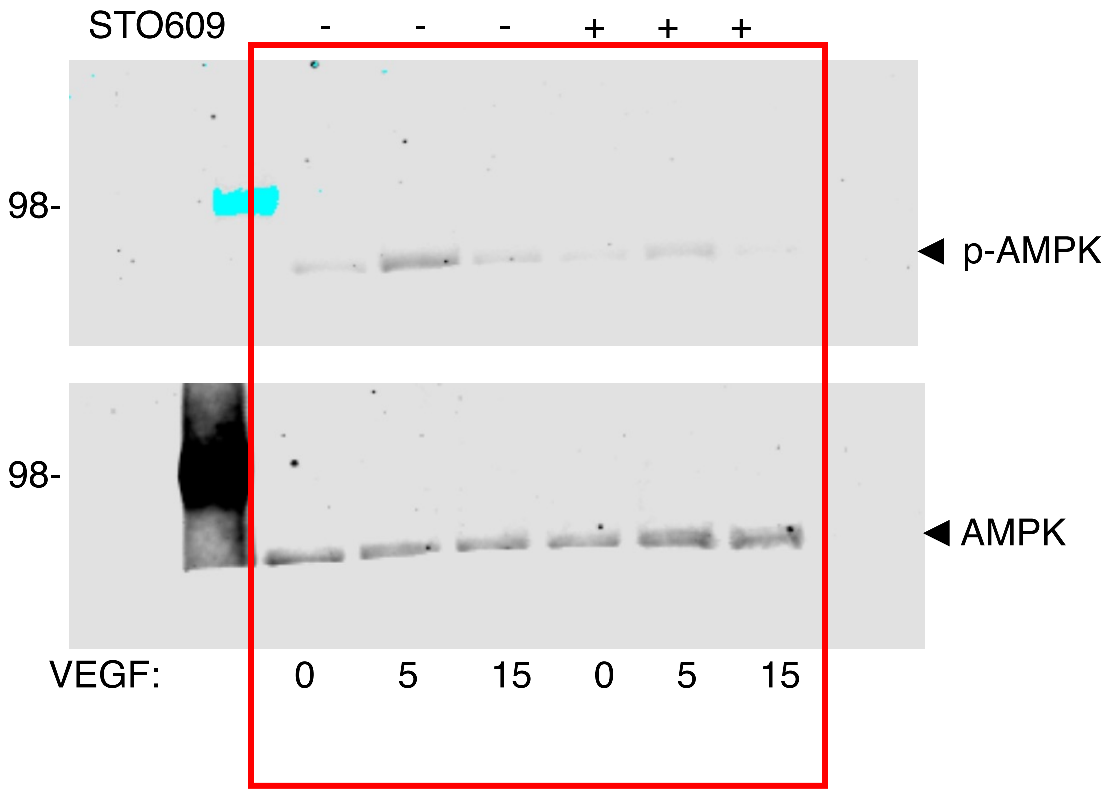

G

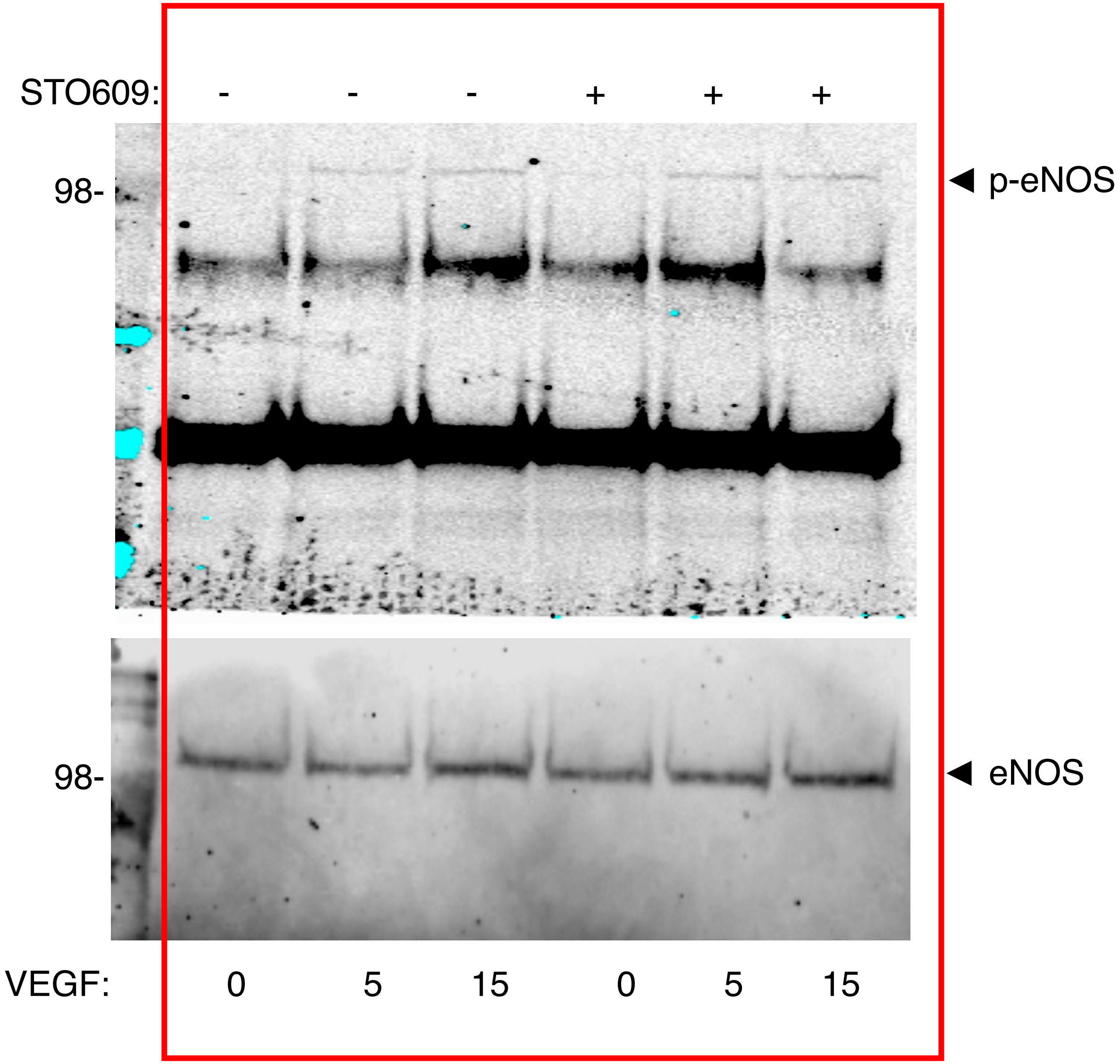

Full unedited gels for Figure 8C

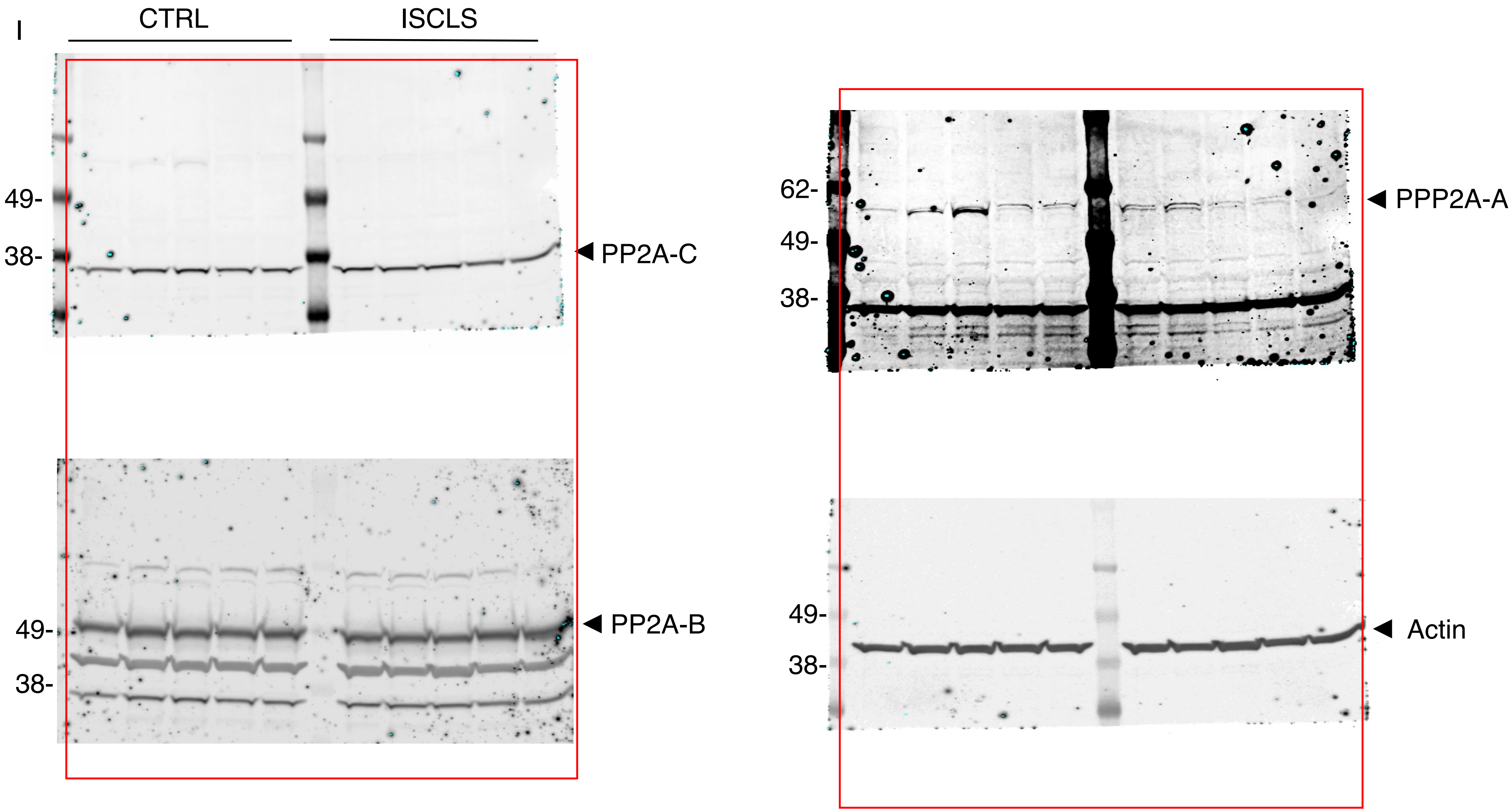

Full unedited gels for Figure S6A

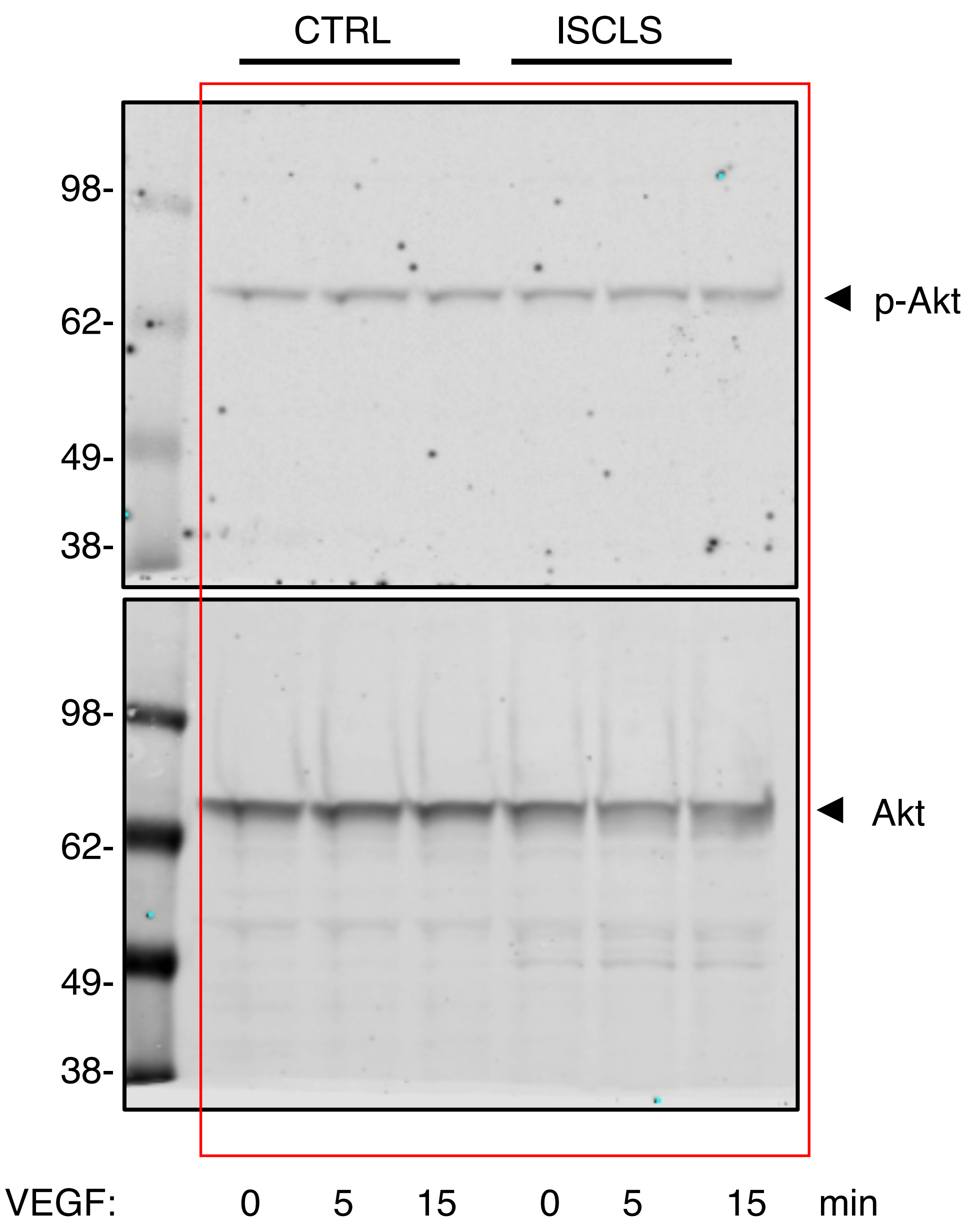

Full unedited gels for Figure 8F, 8J

8F

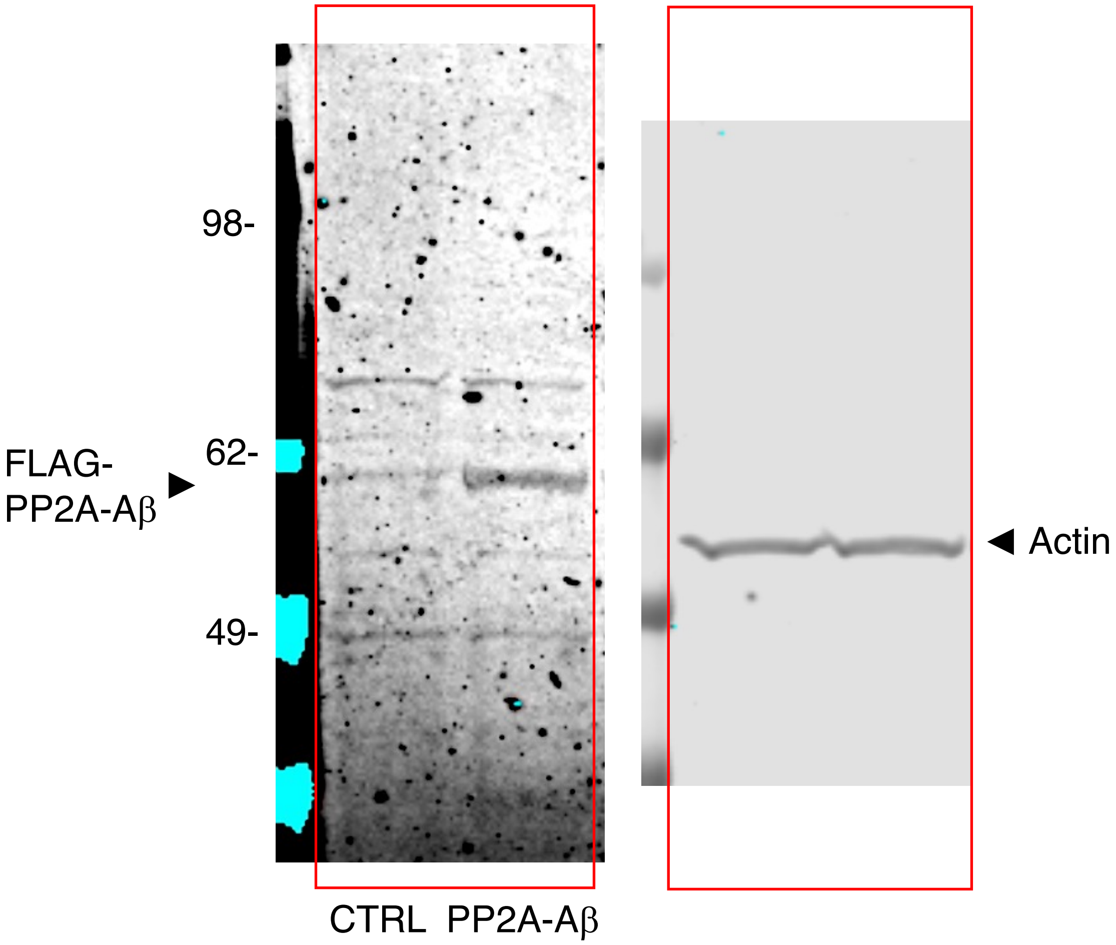

8J

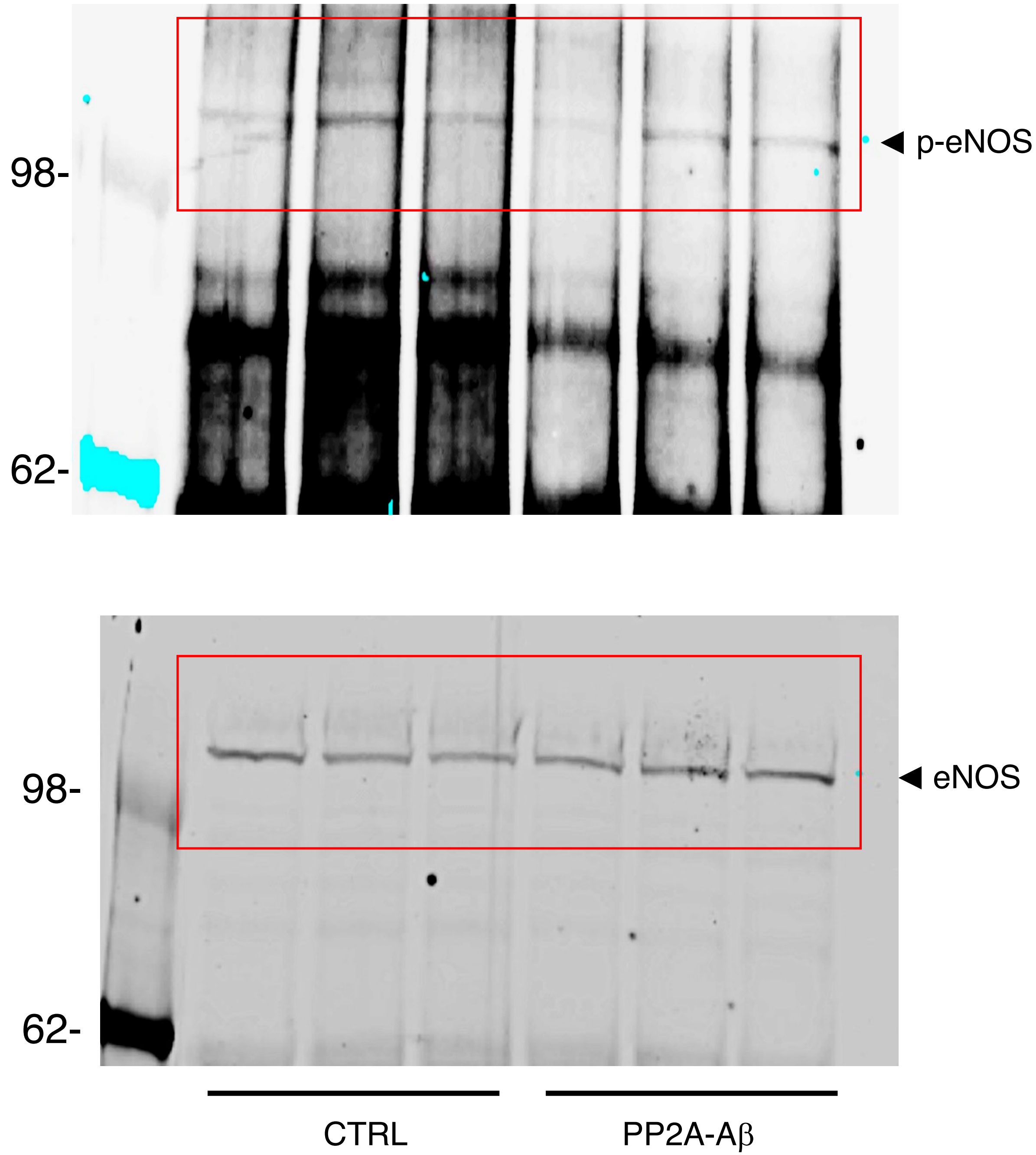

Supplement: Unedited blot and gel images [file jci-134-169137-s152.pdf]
